# Climate change and sexual and reproductive health: A mixed methods scoping review of possible mechanisms

**DOI:** 10.1016/j.isci.2026.117794

**Published:** 2026-10-12

**Authors:** Luissa Vahedi, Susan Pradhan, Sakina Zaidi, Vanessa Brizuela, Sabina Faiz Rashid, Maha El Adawy, Rana Hajjeh, Michelle Gaffey, Zulfiqar A. Bhutta

**Affiliations:** 1 Centre for Global Child Health, The Hospital for Sick Children, 525 University Avenue, Suite 702, Toronto, ON M5G 2L3, Canada; 2 Human Reproduction Programme, World Health Organization, Avenue Appia 20, 1211 Geneva, Switzerland; 3 BRAC James P Grant School of Public Health at BRAC University, 68 Shahid Tajuddin Ahmed Sarani, Mohakhali, Dhaka 1212, Bangladesh; 4 World Health Organization Eastern Mediterranean Region, Monazamet El Seha El Alamia Street, Extension of Abdel Razak El Sanhouri Street, P.O. Box 7608, Nasr City, Cairo 11371, Egypt; 5 National Coalition of Independent Scholars, 125 Putney Road, Brattleboro, VT 05301, USA

**Keywords:** climate change, SRHR, sexual and reproductive health and rights, gender-based violence, LMICs, scoping review, LMIC, mechanism, pathway, women and girls

## Abstract

This scoping review synthesized empirical evidence on the relationships and possible pathways through which climate change could impact sexual and reproductive health and rights (SRHR) in low- and middle-income countries (LMICs). Eligible sources had to investigate climate change and SRHR using any study design. Two independent reviewers conducted screening; data extraction captured exposures, outcomes, associations/relationships, and possible mediators and contextual moderators. From 40,503 database-identified sources, we included 256 peer-reviewed publications across 108 LMICs. We identified 14 potential pathways and developed a typology of mediators and contextual moderators to inform the mechanisms. Five mediator domains were identified: (1) systems and infrastructure, (2) demographic shifts, (3) socioeconomic resources, and (4) behavioral and (5) biological. Six contextual moderator types were isolated: (1) patriarchal norms, (2) cultural power structures, (3) socioeconomic realities, (4) gender-inequitable climate governance, (5) place-based factors, and (6) pre-existing systems/infrastructure. As climate shocks intensify, advancing a mechanistic understanding will be critical to designing responsive, inclusive, and sustainable systems of protection.

## Graphical abstract

**Figure:**
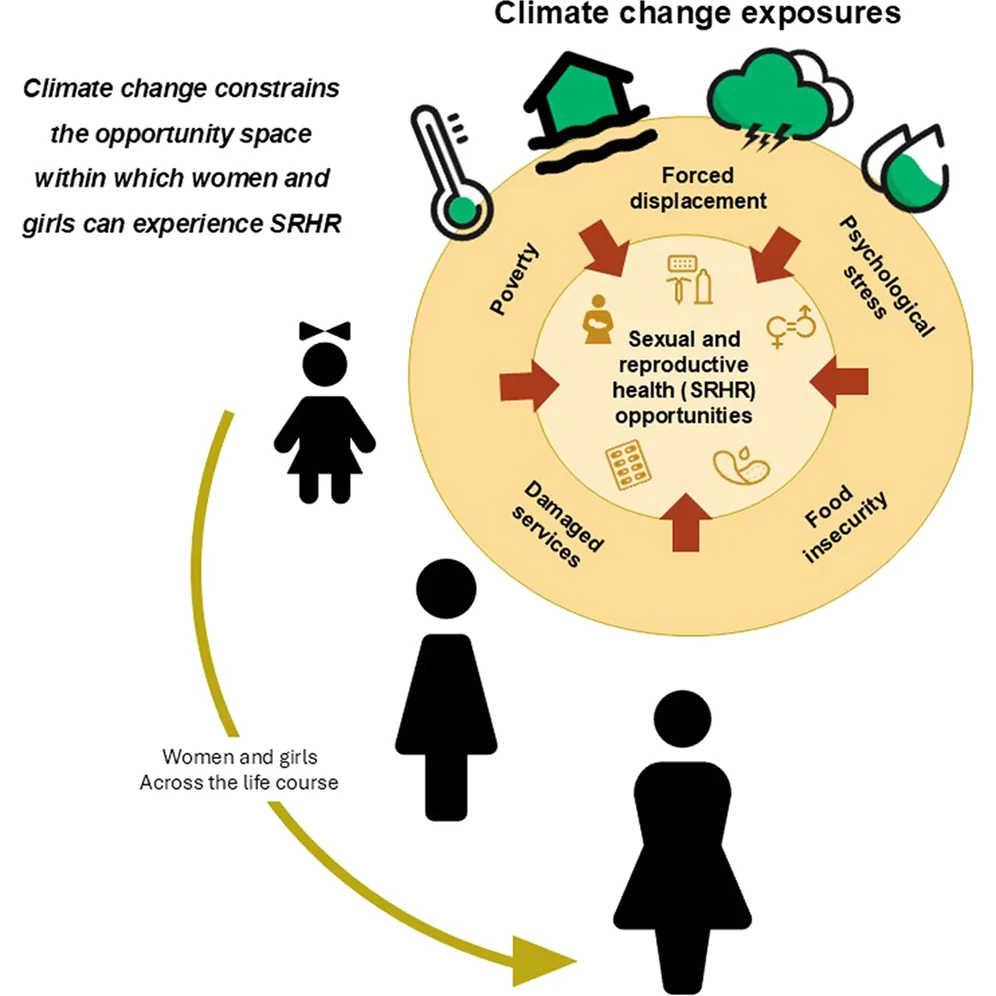

## Teaser

Reproductive medicine; Sexual medicine; Environmental health; Global change

## Introduction

About 3.6 billion people from low- and middle-income countries (LMICs) reside in areas most affected by climate change.^1^ Between 2000 and 2019, extreme weather events (EWEs) (defined as weather events that are rare at a particular place and time of the year such as temperature extremes, floods, heavy precipitation, extreme storms and cyclones, and droughts)^2^ caused an estimated 1.2 million deaths and affected over 4 billion people globally, with the burden disproportionately falling on LMICs.^3^ Further, mortality due to EWE was 15 times higher in LMICs compared to high-income countries.^4^ Climate change is also worsening food and water insecurity, with 2 billion people lacking access to safe water and 770 million, mostly in Africa and Asia, facing hunger due to climate-related disruptions.^4^

One of the most overlooked yet urgent issues concerning the climate crisis are impacts on sexual and reproductive health (SRH) and rights (SRHR) including infrastructure related to empowerment and safety like stable housing, water sanitation and hygiene, and employment.^5^ Health and well-being depend on whether rights like making choices about one’s body, having control over reproduction, and living free from violence are protected and respected.^6^ Globally, SRHR indicators are not on track to achieve Sustainable Development Goal (SDG) 3: Good Health and Well-Being and SDG 5: Achieve Gender Equality and Empower all Women and Girls.

The consequences of climate change on SRHR unfold through various pathways. For example, EWEs and long-term alterations in climate can adversely impact SRHR due to physical healthcare infrastructure damage, disrupted medical supply chains, and forced migration.^7^ EWEs can lead to household-level economic shocks, which are known catalysts for gender-based violence (GBV) and child marriage.^7^^,^^8^ Other harmful pathways may involve biological considerations. For example, rising temperatures can increase the risk of fetal distress, preterm births, low birth weight, and stillbirths.^9^

Despite growing awareness of the linkages between climate change and SRHR adversities, there are gaps in synthesizing the evidence on the underlying pathways of harm within specific contexts, including the mechanisms through which harm unfolds. While several other reviews on climate change and SRHR exist,^8^^,^^9^^,^^10^^,^^11^^,^^12^^,^^13^^,^^14^^,^^15^^,^^16^ existing syntheses are limited in three ways. First, existing reviews tend to synthesize evidence from acute climate disasters like floods or storms in isolation from chronic alternations in climate indicators like temperature.^9^^,^^15^ Second, considering that SRHR is an umbrella term that encompasses a variety of different outcomes, many existing reviews focus on selected SRHR outcomes like maternal aspects of reproductive health,^10^ GBV,^14^ or HIV.^17^ Third, existing reviews tend to summarize the overall relationship between climate change and SRHR (either through meta-analysis or thematic summaries), without sufficient consideration given to classifying mechanisms of harm, context differences, and possible mediating paths.

Building on emerging work focused on empirically investigating harmful pathways,^13^^,^^18^^,^^19^^,^^20^^,^^21^^,^^22^ this mixed methods scoping review strengthens a mechanistic understanding of climate change and SRHR in LMICs. This review organizes the evidence base into distinct pathways. We consider possible mediators (factors affected by climate change that have downstream effects on SRHR) and contextual moderators (pre-existing realities of a particular location or community that modify the effects of climate change on SRHR). Our review includes a range of both acute and chronic climate change exposures and SRHR outcomes. Our work extends beyond biomedical SRHR outcomes to also encompass sociocultural and psychosocial dimensions of SRHR. By including diverse study methods, we can synthesize quantitative associations between climate change and SRHR alongside qualitative evidence of the lived experiences of individuals and communities.

## Methods

The purpose of the review was to explore evidence of relationships/associations between climate change and SRHR in LMICs and elucidate the potential mechanisms underlying those relationships. We used a convergent integrated mixed-methods scoping review design^23^ and adopted the PRISMA ScR guidelines.^24^ No review protocol was registered or published.

*A priori* eligibility criteria were used. Regarding inclusion, studies had to empirically investigate climate change exposures and SRHR outcomes among human populations in LMICs, as defined by the World Bank.^25^ Panel 1 outlines the definitions used to assess climate change exposures and SRHR outcome eligibility. Studies were excluded if they were not peer reviewed and published in non-English languages and before 2000. We included all study designs and methods. Multi-country studies had to have at least one result specific and disaggregated to LMICs.

Panel 1 **Conceptual definitions of climate change and SRHR**

**Climate change**: we defined climate change as alterations in long-term weather patterns due to human activity.^26^ Using an existing three-level framework developed by Bulter in 2024, we conceptualized (1) primary, (2) secondary, and (3) tertiary climate change exposures relative to SRHR.^27^ The primary exposure level included hazardous weather events, both included acute or EWE (heatwaves, storms, floods, droughts, and wildfires) and long-term climatic shifts (rising temperatures, changing precipitation patterns, rising sea levels, glacial melting, and wind speed). The secondary exposure level covered climate change effects on infectious disease pathogen ecology and water quality. The tertiary exposure level included broader human security impacts such as environmental degradation, forced displacement, food and water shortages, political conflict, and famine.^27^ Mental health was considered cross cutting across the primary, secondary, and tertiary effects. We did not include articles that exclusively examined air pollution as this evidence base is considerably larger than other climate change exposures, is less geographically diverse, and more narrowly focused on infant health outcomes (like pregnancy loss and birth anthropometry).^28^^,^^29^^,^^30^

**SRHR**: defined as living in a state of physical, emotional, mental, and social well-being in relation to sexuality and reproductive functions, rather than merely the absence of disease or infirmity. We further operationalized SRHR based on the WHO framework on sexual health and its linkages to reproductive health,^31^ which conceptualizes SRHR as interrelated with human rights and autonomy and identifies several domains corresponding to specific SRHR outcomes.^31^ In addition to the 8 SRHR domains included in the WHO framework, we also added menstrual health and hygiene as a ninth domain given its prominence in the evidence base retrieved and relationship between gender discrimination, stigma, and menstruation:

1. **Antenatal, intrapartum, and postnatal health (which we refer to as maternal-infant health outcomes):** encompasses pregnancy, labor, and delivery outcomes, related to both women and their infants, including stillbirth, miscarriage, birth anthropometry (i.e., birth weight and length), gestational diabetes and hypertension.
2. **Comprehensive education and information:** refers to the existence of programs and policies that disseminate accurate, age-appropriate, and current information on sexuality, reproduction, and sexual and reproductive health.
3. **Contraception counseling and provision:** involves the availability of a range of accessible, affordable, and acceptable contraceptive methods by skilled providers in quality care settings.
4. **GBV:** includes a variety of harmful behaviors such as intimate partner violence (IPV), non-partner sexual violence, harassment, and violence against women and girls (VAWG). We also included stigma and discrimination based on sexual and gender orientation and harmful practices and traditions such as child marriage.
5. **Fertility and infertility:** encompass outcomes such as time to pregnancy, biomarkers of fertility/infertility such as hormone levels, and sperm morphology. We also considered the availability of medical services and supports related to fertility (achieving pregnancy, managing infertility challenges, and ability to conceive a child).
6. **HIV and other STIs**: the presence of HIV or STI in addition to strategies and interventions to reduce the transmission of HIV and other STIs.
7. **Abortion**: refers to the availability of medical practices ensuring safe and regulated termination of pregnancy, protecting individuals’ health. Also included are abortion procedures that are not evidence based, safe, or regulated.
8. **Sexual function and psychosexual health**: includes the availability of therapeutic support to improve sexual health, function, and emotional well-being in intimate relationships. Also included were experiences of sexual function and psychosexual health.
9. **Menstrual health and hygiene**: availability of menstrual hygiene products to manage menstrual flow, privacy for changing these products, and access to proper facilities for disposing the used materials.

The literature search was implemented on January 21, 2025. Four databases were selected to cover medical, social science, public health, and interdisciplinary fields: (1) Medline, (2) Embase, (3) Web of Science, and (4) Scopus. Search strategies were customized for each database using keywords and thesaurus-specific terms related to (1) climate change, (2) SRHR, and (3) LMICs (refer to Tables S1–S3 and Text S1).

Covidence was used to manage the article screening process and remove duplicate records before the title/abstract screening phase. During the title and abstract (inter-rater reliability for title and abstract k = 0.82) and full-text screening phases (inter-rater reliability for full-text review k = 0.66), researchers independently assessed sources to determine eligibility. Conflicts were resolved through discussion and group consensus at both screening stages. Data extraction was conducted using structured charting forms depending on whether the article was (1) quantitative, (2) qualitative, or (3) mixed methods. Two research assistants independently extracted information on (1) study characteristics (including climate change exposures of SRHR outcomes), (2) results, (3) mechanisms, and (4) context. Pilot extraction was conducted on 20 articles. A discussion-based team consensus process resolved any differences in the extracted information/data.

Informed by an epidemiological perspective,^32^ we defined mediators as conditions that are worsened by climate change and have harmful downstream effects on SRHR. We separately extracted quantitative (e.g., statistical analyses of mechanisms or related associations, mediation, and mechanistic interaction) and qualitative evidence (e.g., ethnographies, lived experiences, and perceptions) of possible mediation. We also extracted information related to potential contextual moderators. We define context as pre-existing elements of the physical, social, and political environments that are not impacted by climate change but influence the overall relationship between climate change and SRHR. Context can influence the relationship in terms of impact strength and significance. Contextual elements (empirical studied via moderation, effect heterogeneity, thematic analyses or ethnographies describing particularities of a geographical region, culture, and power structures) helped to determine which populations or regions are most and least affected. In this way, contextual moderation tells us under which conditions the underlying causal effects may be occurring.

The synthesis of quantitative, qualitative, and mixed methods studies occurred simultaneously based on the extracted information. We integrated quantitative measures of association (extracting measures of association and abstracting textual descriptions of results), qualitative perceptions, lived experiences, ethnographies, and mixed methods studies to better understand the pathways and underlying mechanisms through which climate change can influence SRHR. We did not privilege one type of evidence over another. Our synthesis focused on breadth and triangulation rather than a hierarchy of evidence. Our integration of quantitative and qualitative evidence informed a conceptual framework that we present in the results.

Extracted information was analyzed using a variety of approaches. First, we summarized the distribution of articles by general characteristics, including funding organizations. Second, we categorized empirical evidence by pathway, wherein a pathway was eligible for reporting in our analysis if it was studied by three separate sources. This approach ensured that the pathways presented were studied in at least three different articles, allowing us to identify patterns in results and gaps, rather than reporting evidence from a single source. From the studies reporting on each pathway, we synthesized extracted information on mediators and contextual moderators and presented these components using flow diagrams, to visualize the underlying mechanisms of harm. In this review, we distinguish between pathways and mechanisms. A pathway refers to a set of at least three articles examining a specific climate change exposure and SRHR outcome pair, whereas a mechanism explains how, why, and under what conditions the harmful relationship between climate change and SRHR occurs.

While our extraction framework was informed by concepts from causal inference, the evidence informing the synthesis varied. Some studies explicitly examined mediation, moderation, interaction, effect heterogeneity, or stratification through quantitative analyses. Other studies, particularly qualitative and mixed methods research, described perceived pathways and contextual conditions through participant lived experiences and interpretations. The resulting typology should, therefore, be interpreted as a synthesis of empirically tested relationships, evidence-informed explanations, and hypothesized pathways derived from integrating findings across studies (refer to Table 1) rather than as a compilation of formally established causal mechanisms.

**Table 1:** Evidence map of climate change and SRHR pathways in LMICs, organized by outcome

Pathway	Evidence base	Evidence type	Mechanistic details	Illustrative contextual examples
EWEs (floods/cyclones) → VAWG	30 (6 quant, 15 qual, 9 mixed)	Quant + qual + mixed	Acute EWE can: • Increase risk factors for violence perpetration within the home (e.g., economic shocks, property loss, unemployment, and substance use among men) • Lead to forced displacement into overcrowded emergency camps, which can encourage opportunistic violence • Promote economic outmigration of men and boys and their influx into host communities, increasing VAWG risk	Qualitative work in Bangladesh displacement shelters reported that women/girls experiences harassment and fear^33^; research conducted in Uganda, Zimbabwe, and Mozambique showed greater IPV odds after severe weather^34^; Kenyan research linked flood frequency to physical IPV.^35^
Drought/water insecurity → VAWG	13 (7 quant, 5 qual, 1 mixed)	Quant + qual + mixed	Droughts/water insecurity can lead to: • Agricultural disruption and male unemployment and related alcohol use, which are risk factors for violence perpetration in the home • Heightened domestic burdens among women/girls related to water fetching (including longer travel and wait times for water) • Increased exposure to opportunistic abusers while fetching water over longer distances • Household conflict concerning the gender-inequitable use of water	Quantitative study conducted in India linked drought-induced male unemployment to VAW^36^; Zimbabwe-based quantitative study documented violence while fetching water^37^; Tanzanian study reported associations between water insecurity and IPV^38^; qualitative work in Kenya described household conflict over water.^39^
Rainfall shocks/variability → VAWG	6 (3 quant, 3 qual)	Quant + qual	Rainfall shocks can: • Reduce agricultural productivity, increase economic/employment insecurity and poverty-related emotional stress, and promote male alcohol consumption, all of which are risk factors for VAWG • Reduce women/girls’ empowerment while increasing men’s control over decision-making and movement • Heighten women/girls’ domestic burdens and time spent confined to the home, exposed to abusers	Quantitative study from Peru linked dry shocks with physical IPV^40^; Indonesian study reported a harmful link between the standardized precipitation and evaporation index and domestic violence^41^; Nicaraguan qualitative study described women/girls as ,”prisoners in their homes” during heavy rains, thereby intensifying traditional gender norms and IPV.^42^
EWEs (floods/cyclones) → child marriage	12 (5 quant, 6 qual, 1 mixed)	Quant + qual + mixed	Climate disasters pose 3 shocks that can have downstream impacts on child marriage: • Agricultural disruption and lack of employment opportunities, reducing household resources • Educational disruptions • Forced displacement to emergency shelters, where opportunities for women and girls are more limited in terms of education, employment, and movement and exposure to abusers is more likely. In some cases, experiences of harassment or sexual violence precipitate child marriage to protect girls’ perceived honor. Child marriage was used as a household coping mechanism against extreme weather event resource constraints.	Bangladesh/India/Nepal/Pakistan quantitative analysis linked extreme wet conditions with child marriage;^43^; qualitative research from Ghana described marriage as a coping strategy to reduce household resource burdens after flooding.^44^
Droughts → child marriage	11 (3 quant, 5 qual, 3 mixed)	Quant + qual + mixed	Droughts have the potential to reduce agricultural yield/crop failure, culminating in household economic shocks/food insecurity. In situations of drought, parents and male family members may migrate in search of economic opportunities, leaving women and girls abandoned and socially vulnerable. Child marriage operates as a survival strategy to reduce household resource burdens, achieve socioeconomic stability, or ameliorate climate-intensified domestic labor like traveling large distances in search of water.	A South Asia analysis (focused on Bangladesh, India, Nepal, and Pakistan) linked drought indicators with child marriage, especially in Bangladesh and coastal Andhra Pradesh^43^; qualitative work from Chad described famine, male exodus, abandonment, and dowry dynamics within the context of droughts.^45^
Droughts → HIV/AIDS	13 (8 quant, 3 qual, 2 mixed)	Quant + qual + mixed	Mechanisms involved agricultural disruption, impaired health behaviors, and reduced access to health systems. Droughts induced economic, food, and water insecurity, posing consequences for HIV-related medical care (ART adherence and medical care access) and malnutrition (increased food prices, hunger, and reduced diet quality). Regarding nutritional consequences, HIV risk behaviors such as transactional sex and related decreased contraceptive emerged as survival strategies for food insecurity and hunger.	South African evidence showed retention HIV care declines during drought and qualitatively described water scarcity affecting medication adherence^46^; Lesotho, Tanzania, Uganda, and Zambia quantitatively linked drought poverty with high-risk sex among rural women.^47^
EWEs (floods) → HIV/AIDS	6 (2 quant, 4 qual)	Quant + qual	Floods have the potential to: • Damage transportation, healthcare, and WASH infrastructure, leading to medication supply chain issues, ART disruptions, and barriers to HIV care • Create agricultural disruptions and economic hardships, culminating in food insecurity and migration/forced displacement, thereby disrupting access to care and ART adherence • Incentivize transactional sex as a hunger survival strategy, leading to HIV incidence	Sub-Saharan Africa analysis linked heavy rainfall with HIV infection odds^48^; Malawi healthcare providers described ART stockouts, damaged medications, flooded roads, and increased replacement demand.^49^
EWEs (floods/cyclones) → maternal-infant outcomes	37 (20 quant, 9 qual, 6 mixed)	Quant + qual + mixed	This mechanism focused on (1) disaster-induced damage to health, water/sanitation, and transportation infrastructure; (2) forced displacement; and (3) food insecurity, all of which have the potential to impose downstream impacts on postpartum care (i.e., reduced access and quality care), facility births, ANC use and accessibility, pregnancy loss, neonatal mortality, and low birth weight.	A Mozambique cyclone was associated with reduced ANC^50^; India flood exposure reduced PNC^51^; a multi-country study (Bangladesh, India, Jordan, and Pakistan) noted that flood exposure during pregnancy was associated with increased pregnancy loss^52^; qualitative evidence from South Sudan described how pregnant women cannot swin in flooded areas to attend ANC.^53^
Droughts → maternal-infant outcomes	12 (9 quant, 3 qual)	Quant + qual	Droughts have the potential to induce water scarcity and agricultural disruptions, both of which magnify women’s domestic labor, emotional distress, and food insecurity, potentially leading to low birth weight and length. During drought periods, elevated temperatures impose transportation burdens, reducing post-natal care access.	Tanzania linked insufficient rainfall and poor harvest interactions with lower birth weight and length^54^; Sierra Leone linked contemporaneous gestation drought with LBW^55^; qualitative research from Kenya described heat-related transportation barriers to postpartum care.^56^
EWEs (floods/cyclones) → contraception	7 (2 quant, 3 qual, 2 mixed)	Quant + qual + mixed	Climate disasters can lead to (1) physical infrastructure damage of health and transportation systems and (2) forced displacement. These intermediaries may negatively impact contraception use and accessibility as well as the desire for more children and son preferences in some regions.	Descriptive quantitative research in Bangladesh reported son preference during EWEs^57^; India linked climate vulnerability with lower modern contraceptive use^58^; qualitative evidence from Nepal described lack of contraceptive access during EWEs, leading to unintended pregnancy.^59^
EWEs (floods/cyclones) and salinity → menstrual health and hygiene	13 (1 quant, 11 qual, 1 mixed)	Mostly qual + limited quant	Floods, rising sea levels, increased evaporation, & salinization can lead to: • Damaged health facilities and water and sanitation infrastructure, limiting access to water & supplies • Forced displacement to emergency camps with limited privacy, which is important for menstrual health and hygiene in contexts where menstruation is stigmatized • Barriers to accessing menstrual hygiene products • Impaired menstrual hygiene practices that require access to clean water • The feminization of poverty given that economic shocks promote women’s precarious labor and reduced purchasing power	Descriptive quantitative evidence from Pakistan reported limited SRH services/sanitary kits and inadequate sex-separated latrines in displacement camps^60^; qualitative evidence from Bangladesh described internalized stigma (shame) and difficulty washing/drying menstrual clothes during floods.^61^
Temperature → maternal-infant outcomes	90 (89 quant, 1 qual)	Mostly quant	Physiological responses to high-temperature exposure such as compromised thermoregulation, oxytocin secretion, and dehydration were linked to outcomes such as neonatal mortality, pregnancy loss, and low birth weight.	A multi-country study in East Africa reported associations between heat exposure and post-neonatal (between 28 days and 1 year) mortality^62^; Southern Asia and Eastern and Western Africa showed associations between heat exposure and LBW odds, compared to Western Africa^63^; Iran linked extreme temperatures (hot and cold) with preterm birth^64^; qualitative research from Gambia described experiences of heat strain during pregnancy.^65^
Temperature → fertility/family planning preferences	12 (11 quant, 1 qual)	Mostly quant	Exposure to high temperatures can negatively affect sperm morphology, count, concentration, and motility as well as semen volume. Exposure to high temperatures may also affect fertility preferences among couples, thereby affecting the total fertility rate or the sex balance of offspring.	Research from India reported that high temperature was associated with reduced progressive sperm motility^66^; other research in LMICs reported that multiple heatwaves were associated with lower sperm number and morphology^67^; qualitative evidence from Ethiopia described families limiting births to cope with changes in climate.^68^
Salinity → gestational HTN	3 (3 quant)	Quant only	Climate disasters like floods, rising sea levels, and increased evaporation can lead to the salinization of water sources. The salination of ground water increases vulnerability to hypertensive disorders during pregnancy. Ground water use from shallow & deep tube wells can increase systolic and diastolic blood pressure and urinary sodium levels. The increased dietary sodium intake, due to salination and the type of water sources available, is related to pre-eclampsia and gestational hypertension.	Quantitative evidence from Bangladesh indicates that women consuming water from tube-well (groundwater) were at highest risk of pre-eclampsia/gestational hypertension compared to rainwater users and a dose-response relationship between water sodium concentration and disease odds^69^; no qualitative sources were identified.

VAWG, violence against women and girls; IPV, intimate partner violence; ART, antiretroviral therapy; WASH, water, sanitation, and hygiene; ANC, antenatal care; PNC, postnatal.

## Results

A total of 40,503 articles were identified by the search. Duplicates (*n* = 7,129) from the database-identified sources were removed, culminating in 33,474 articles for screening. Following the title and abstract screening, the full texts of 1,942 articles were assessed for eligibility. A further 1,686 articles were excluded during the full text review for reasons such as not reporting a climate change exposure or an SRHR outcome or not using an empirical study design. Finally, a total of 256 studies were included in the scoping review. Figure 1 summarizes the study selection process.

**Figure 1:**
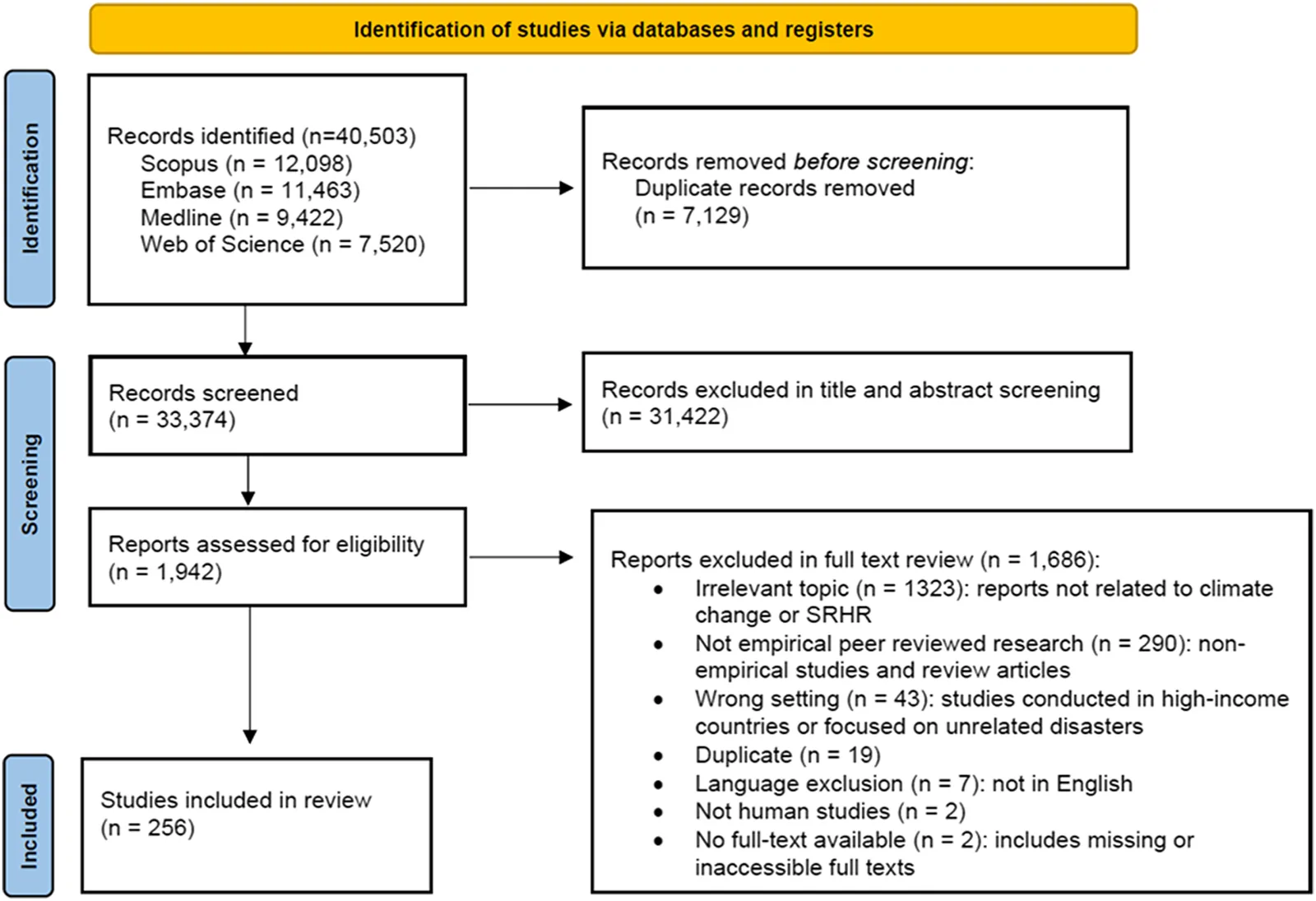
PRISMA flow diagram

### General study characteristics

Most studies employed quantitative methods (*n* = 173), followed by qualitative methods (*n* = 55), and lastly mixed methods, which combined both qualitative and quantitative approaches (*n* = 28). Figure 2 illustrates the geographical distribution of funding organizations and study settings. Various funding organizations were disclosed among the included studies (national governments, internal university grants, and private foundations), 70% of which originated from three countries: United States (*n* = 65), China (*n* = 66), United Kingdom (*n* = 40), and Australia (*n* = 16). Other notable funders were Norway (*n* = 9), Canada (*n* = 9), Germany (*n* = 7), and India (*n* = 5). Of the total identified funders, 34% were governments bilaterally funding research in other LMICs, 27% were LMIC governments domestically financing their own research, 12% were internal university grants, 10% were sourced from philanthropies, 5% received no funding, and 3% were from multilateral international organizations. Most research studies were conducted in China (*n* = 59), Bangladesh (*n* = 46), India (*n* = 27), Uganda (*n* = 28), Pakistan (*n* = 25), Malawi (*n* = 24), and Kenya (*n* = 24).

**Figure 2:**
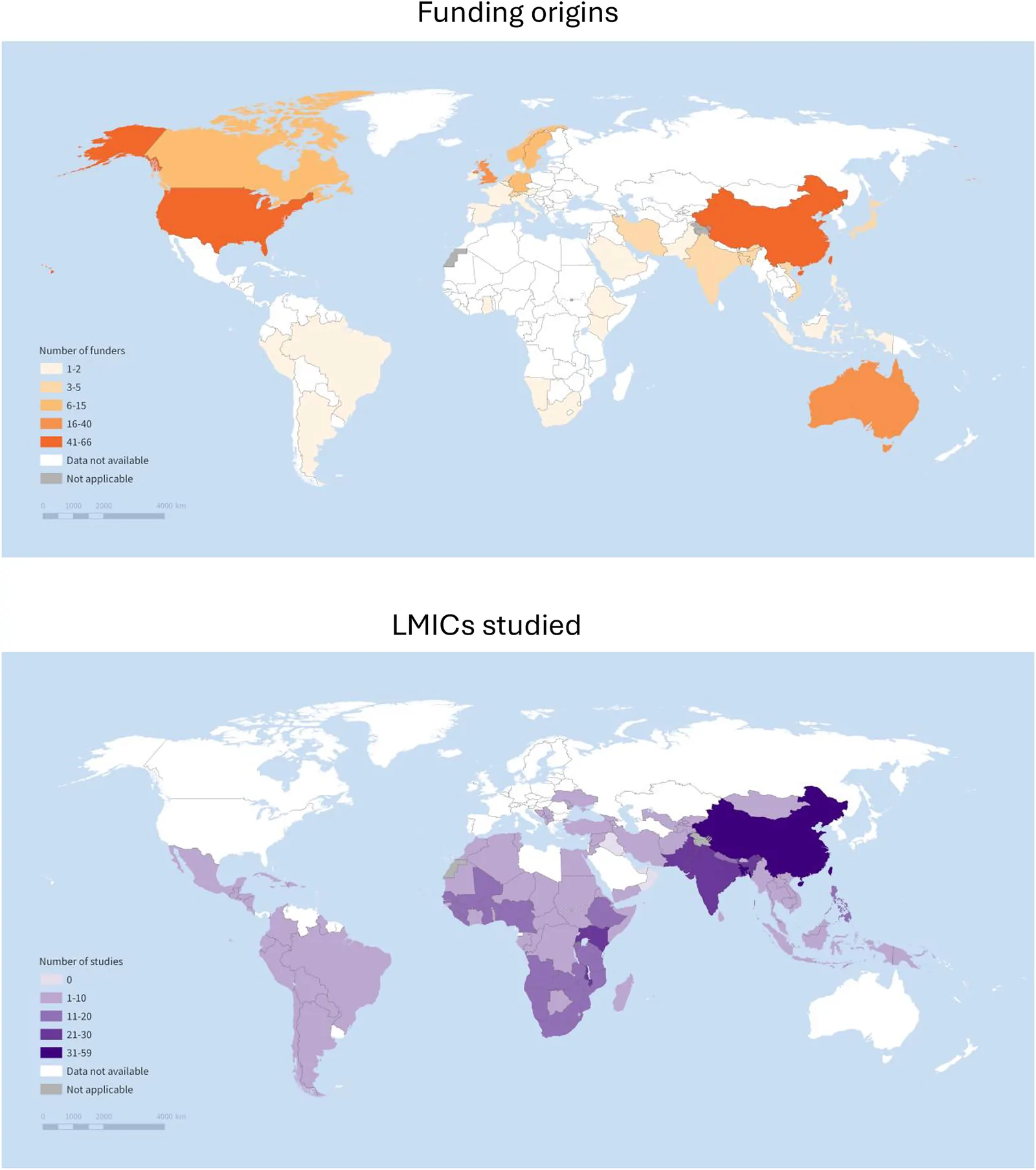
Geographical distribution of funders and settings

Of the climate change exposures studied (panel 1), primary exposures were the most commonly reported: temperature/heat effects (raising temperatures over time and temperature shocks) (*n* = 125), floods (*n* = 79), droughts (*n* = 57), storms (cyclones and hurricanes) (*n* = 32), precipitation (in excess or lack thereof) (*n* = 27), humidity (*n* = 8), and wildfires (*n* = 4). Regarding the secondary forms of climate change affecting water sources, water insecurity (*n* = 11), salinity (*n* = 11), and sedimentation (*n* = 10) were studied. Lastly, climate change-related famine and hunger were the least studied (*n* = 2). In these studies, climate change shocks (like droughts and floods) were identified as structural drivers of food insecurity and hunger, given diminished access to water and land and increased competition from non-food agricultural product use.^70^ Famine and hunger were also described as push factors for climate-induced forced migration.^71^

Based on our adapted WHO SRHR framework, maternal-infant outcomes (including pregnancy loss, birth anthropometry like weight and length, and preterm birth) were most studied (*n* = 160), followed by GBV and harmful practices (e.g., child marriage and female genital cutting) (*n* = 78), fertility (*n* = 28), HIV, and STIs (i.e., prevalence, treatment access, and adherence) (*n* = 26). Lesser-studied SRHR domains were contraception (*n* = 18), menstrual health and hygiene (*n* = 12), sexual function and psychosexual health (*n* = 5),^72^^,^^73^^,^^74^^,^^75^^,^^76^ and abortion (*n* = 2).^77^^,^^78^ No studies investigated outcomes related to comprehensive education and information.

### Synthesis of pathways

The 256 included studies were grouped into 14 distinct pathways involving 6 climate change exposure types (rainfall shocks/variability, floods, drought and water insecurity, storms, salinity, and temperature) and 8 SRHR-related outcomes (VAWG including IPV and sexual violence; child marriage; menstrual health and hygiene; contraception; gestational hypertension; maternal-infant outcomes like pregnancy loss, preterm birth, low birth weight, and post-partum health; fertility; and HIV/AIDS).

Our synthesis incorporates both empirically tested mechanisms, measures of association indicating the direction and significance of relationships, and evidence-informed pathways suggested by qualitative and mixed methods research. The quantitative evidence was concentrated on estimating associations between maternal-infant outcomes and (1) temperature changes/shocks and (2) floods as well as associations involving drought and GBV/harmful practices. The qualitative evidence was concentrated on pathways leading to menstrual health and hygiene, drawing on lived experiences of adolescent girls affected by climate disasters like floods and cyclones and salinization.

Figure 3 illustrates the typology of exposure-outcome pairs that delineate the pathways detailed in Table 1. Most relationships and pathways involved EWEs, noting how these damage infrastructure,^49^^,^^59^^,^^60^^,^^74^^,^^79^^,^^80^^,^^81^^,^^82^^,^^83^^,^^84^ may result in displacement to ill-equipped emergency camps,^33^^,^^72^^,^^75^^,^^79^^,^^81^^,^^85^^,^^86^^,^^87^^,^^88^^,^^89^ increase household economic stress^33^^,^^40^^,^^74^^,^^88^^,^^90^^,^^91^^,^^92^^,^^93^^,^^94^^,^^95^^,^^96^^,^^97^ and food insecurity,^46^^,^^48^^,^^74^^,^^84^^,^^87^^,^^91^^,^^95^^,^^96^^,^^97^^,^^98^^,^^99^^,^^100^^,^^101^^,^^102^^,^^103^^,^^104^^,^^105^^,^^106^ further entrench gender-inequitable norms concerning household labor,^42^^,^^79^^,^^90^^,^^107^^,^^108^^,^^109^^,^^110^ and reduce opportunities for healthy coping via substance use.^34^^,^^35^^,^^40^^,^^79^ These intermediaries pose downstream consequences for SRHR outcomes such as VAWG,^33^^,^^34^^,^^35^^,^^36^^,^^37^^,^^39^^,^^42^^,^^61^^,^^74^^,^^75^^,^^79^^,^^80^^,^^87^^,^^88^^,^^89^^,^^91^^,^^108^^,^^111^^,^^112^ child marriage,^43^^,^^44^^,^^45^^,^^94^^,^^95^^,^^96^^,^^97^^,^^103^^,^^104^^,^^106^^,^^109^^,^^113^^,^^114^^,^^115^ HIV/AIDS transmission and reduced care,^47^^,^^48^^,^^49^^,^^70^^,^^74^^,^^75^^,^^84^^,^^100^^,^^107^^,^^116^^,^^117^^,^^118^^,^^119^ maternal-infant disparities^51^^,^^59^^,^^60^^,^^74^^,^^80^^,^^82^^,^^83^^,^^88^^,^^90^^,^^120^^,^^121^^,^^122^^,^^123^^,^^124^^,^^125^^,^^126^ like low birth weight and pregnancy loss,^52^^,^^54^^,^^55^^,^^123^^,^^126^^,^^127^^,^^128^^,^^129^ reduced contraception access,^58^^,^^59^^,^^60^^,^^75^^,^^80^^,^^130^ and impaired menstrual health and hygiene.^60^^,^^61^^,^^72^^,^^74^^,^^75^^,^^79^^,^^81^^,^^85^^,^^86^^,^^105^^,^^120^^,^^131^

**Figure 3:**
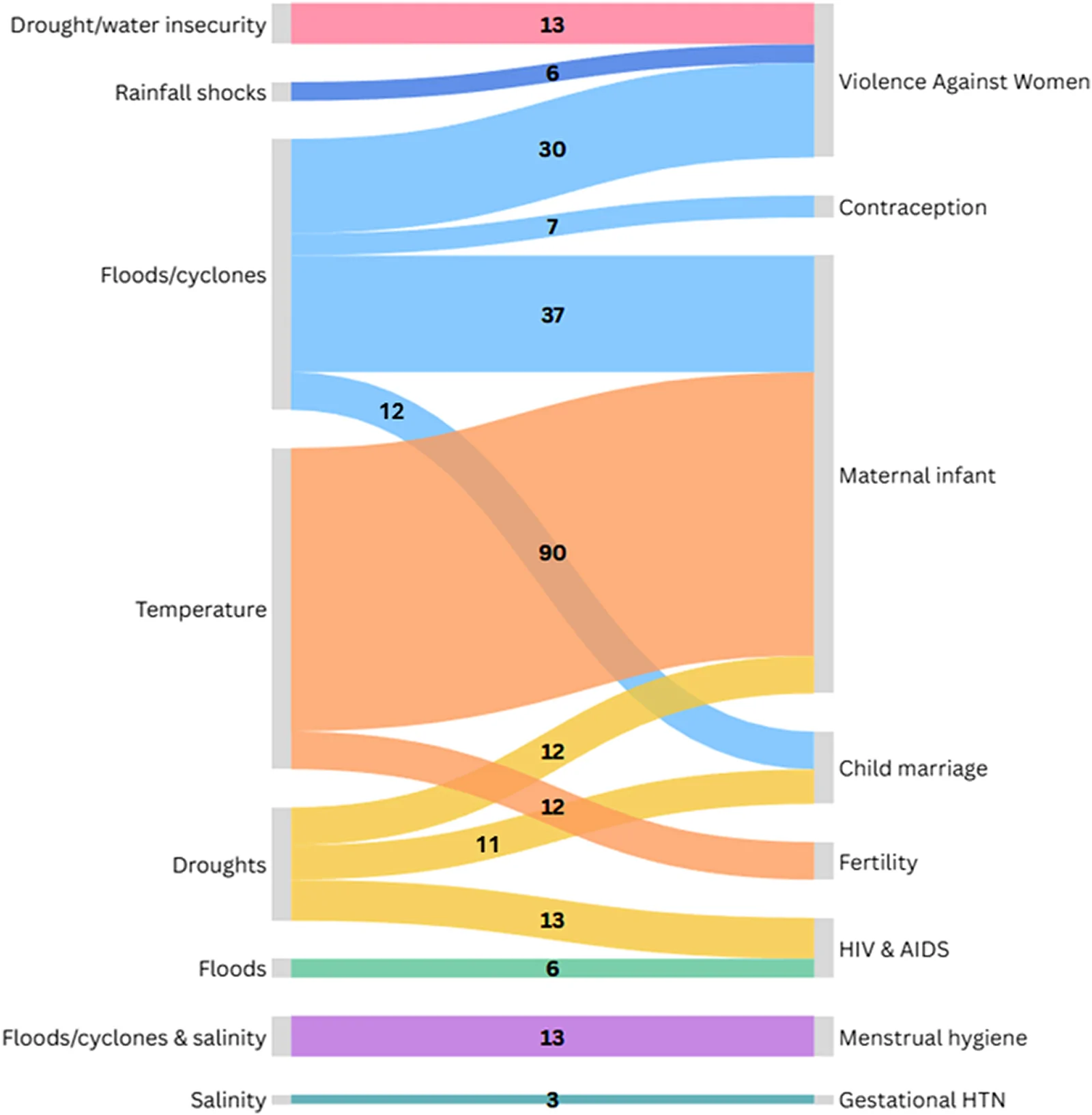
Typology of exposure-outcome pairs investigated across all study designs

Regarding chronically altered climate indicators, most pathways involved biological and physiological changes involving pregnancy and fertility. Temperature was the most frequently studied indicator that was tracked. However, ambient temperatures were tracked longitudinally during pregnancy with less attention given to alterations in temperature over multiple decades. This evidence was linked to maternal-infant outcomes in a lagged fashion, wherein maternal exposure to high and low temperatures during specific gestational periods was associated with adverse maternal-infant outcomes like pregnancy loss.^132^^,^^133^^,^^134^^,^^135^^,^^136^^,^^137^^,^^138^^,^^139^^,^^140^^,^^141^^,^^142^^,^^143^ Other outcomes studied in relation to increased temperatures were also fertility (sperm quality and morphology),^66^^,^^67^^,^^68^^,^^144^^,^^145^^,^^146^^,^^147^^,^^148^^,^^149^^,^^150^ violence against women/girls (such as IPV),^151^^,^^152^ contraception,^147^^,^^153^ and menstrual disorders.^154^ The salinization of water sources is also a slow-onset climate change hazard quantitatively studied in relation to maternal-infant outcomes like gestational hypertension and pre-eclampsia.^69^^,^^155^^,^^156^ In qualitative studies, salinity and related exposures (like river bank erosion, sea level rise, and saltwater intrusion) were investigated in relation to EWEs (such as floods) making it difficult to parse out relationships involving salinity (a slow-onset event) and EWEs.^95^^,^^96^^,^^110^^,^^113^^,^^120^^,^^157^^,^^158^ The qualitative literature made linkages between climate-affected areas (due to a constellation of salinity and other hazards) and gender inequities disproportionately affecting women/girls related to empowerment, decision making, menstrual health and hygiene, violence, and harmful practices like child marriage.^74^^,^^95^^,^^106^^,^^110^

### Conceptual model of mechanisms involving possible mediators and contextual moderators

To synthesize findings, we developed a conceptual model illustrating our typology of mediators and contextual moderators (refer to Figure 4). The mediators are situated conceptually on the hypothesized causal pathways between climate and SRHR and can be grouped into 5 domains, namely, (1) systems and infrastructure, (2) demographic shifts, (3) economic resources, and (4) coping behaviors and (5) biology. The systems and infrastructure domain includes damage to physical infrastructure (transportation, healthcare, water sanitation and hygiene, and housing) and system disruption (dysregulated systems of safety/rule of law, education, health, supply chains related to menstrual hygiene, medication, contraception, etc.).

**Figure 4:**
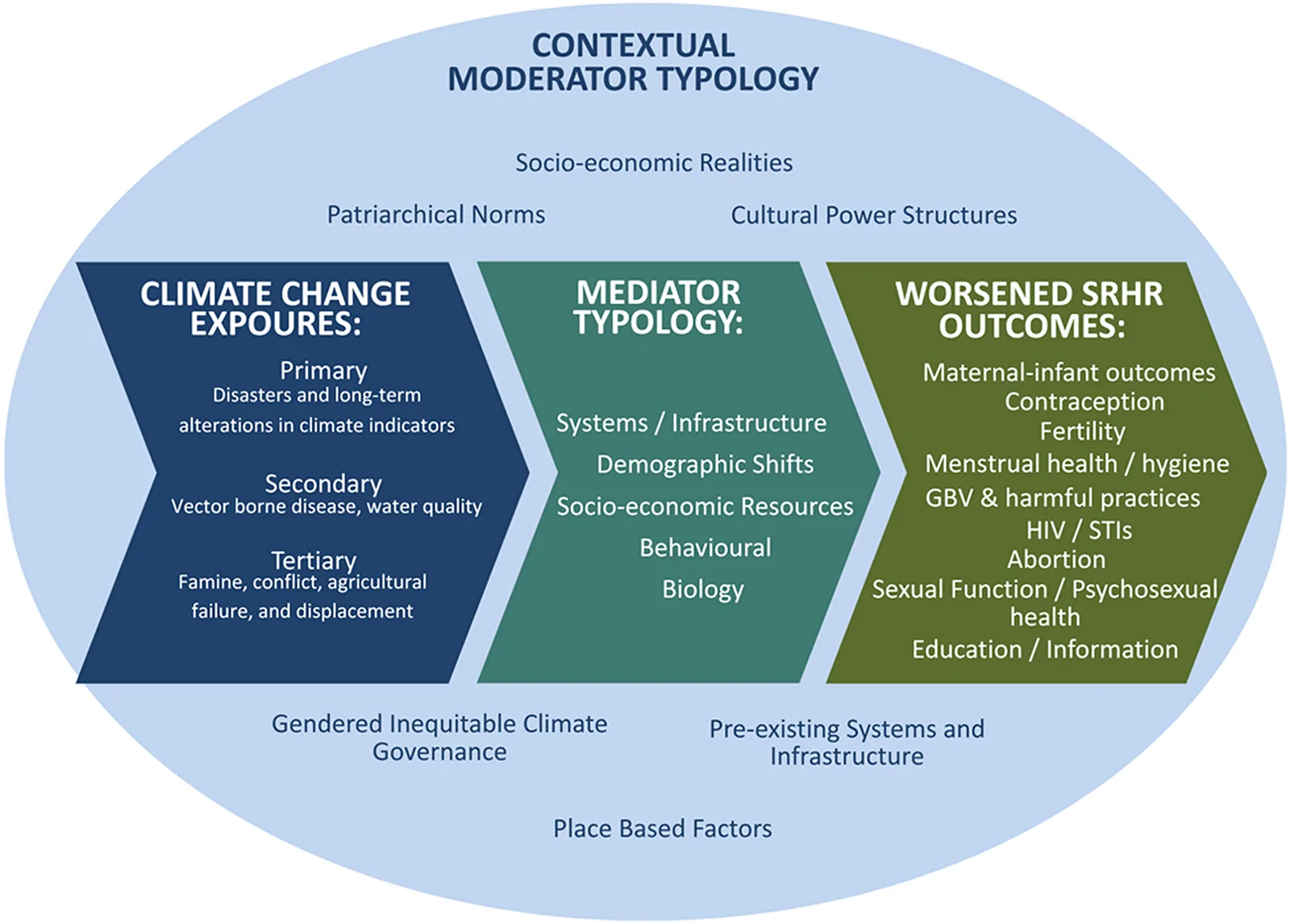
Conceptual model linking climate change and SRHR in LMICs

Physical damage to (1) healthcare and (2) transportation infrastructure, and (3) health system disruption were most studied. The pathways that investigated system damage and disruption involved EWE like floods and storms and SRHR outcomes that require access to health supplies (medication, contraception, and menstrual hygiene products) or healthcare providers (i.e., ante- and postnatal care, facility births, and HIV/AIDS treatment and counseling). For example, when discussing the impact of floods, healthcare providers from Malawi described antiretroviral therapy (ART) stockouts, damaged medications, flooded roads, and increased demand for replaced medication.^49^ Further, qualitative evidence from Nigeria noted that rainstorms flooded local communities, sweeping raw sewage and urine into homes.^90^ This contaminated water and instruments used during home deliveries.^90^ As a result of exposure to unhygienic and unsafe environmental conditions in the first minutes of life, participants recounted newborn children who developed neonatal tetanus infection and died shortly after.^90^

The demographic shift domain involves movement and migration due to forced displacement (internal and external) and male outmigration as mediators, both of which can result in family separation, loss of social support, and confinement in the home or emergency shelters, with implications for SRHR. For example, male outmigration often increases domestic responsibilities for women and girls, who are left to manage household tasks and care for family members. This dynamic reinforces and deepens existing gender inequities. Commonly studied was displacement to emergency shelters/camps, appearing in half of the pathways. Male outmigration (referring to displacement of men/boys and their influx into host communities) and family separation, loss, and reduced social support each appeared in over one-quarter of the pathways studied. Male out migration and family separation can increase the risk factors for different forms of GBV like sexual violence and sexual harassment as well as child marriage. For example, in a qualitative study conducted in Chad, a local women’s advocate observed that drought-induced famine resulted in men fleeing in “exodus,” resulting in familial abandonment and men looking to marry young girls from poor families.^45^

The socioeconomic resources domain encompasses both economic shocks (magnifying unemployment, precarious labor domestic/parenting burdens, and reducing agriculture, livestock, and fishing yield) and food and water insecurity, which reduce diet quality and increase hunger, distance to water sources, and household water conflicts. Economic/employment shocks, agricultural/fishing shocks, food insecurity, and hunger each appeared in over half of the pathways identified. Household economic shocks were reported as precipitating factors for violence perpetration and the use of child marriage as a coping mechanism as well as reduced healthcare seeking in the pre- and postnatal periods. For example, quantitative evidence linking drought and child marriage was strongest in Bangladesh and coastal Andhra Pradesh in South India (regions where household resources are sensitive to drought).^43^ A qualitative study from Ghana described child marriage as a household coping strategy during climate-related resource scarcity.^44^ Further, food and water insecurity were related to reduced HIV treatment adherence and to sexual risk behaviors that can increase the risk of HIV transmission. In South Africa, water scarcity disrupted ART adherence by constraining the ability to ingest medication.^118^

Mediators in the behavioral coping category include an increase in potentially harmful behaviors like substance use, transactional sex, and multiple sex partners as well as reduced healthcare seeking, empowerment, and mental and emotional health. Mediators studied in over one-third of the pathways were (1) deteriorated mental and emotional well-being and (2) men’s substance use (mainly alcohol). Deteriorated mental/emotional well-being and substance use among men appeared in the mechanisms between climate change and domestic violence/ IPV. For example, qualitative evidence from Kenya described household conflict and emotional distress arising from water insecurity and associated domestic burdens.^39^ In Bangladesh, qualitative research following cyclones documented emotional distress and heightened sexual harassment among women and girls in emergency shelters.^33^ A study from India linked drought-induced male unemployment to increased violence against women.^36^ Multi-country evidence from Lesotho, Tanzania, Uganda, and Zambia suggested that drought-related poverty and food insecurity increased engagement in high-risk sex behaviors among rural women.^47^

Reported mediators situated at the biological level are physiological processes related to thermoregulation, oxytocin release and uterine contraction, intrauterine growth restriction, sperm count and morphology, fetal heart rate and movement, and sodium intake during pregnancy. Mechanisms involving biological mediators investigated maternal-infant outcomes (pregnancy loss, pre-term birth, low birth weight, gestational hypertension) and fertility. Biological mediators were most apparent in studies examining temperature and salinity exposures. For example, studies from Gambia,^65^ Iran,^64^ Ethiopia,^62^^,^^68^ and other LMIC settings^63^^,^^66^^,^^67^ linked heat exposure during pregnancy to physiological processes associated with pregnancy loss, preterm birth, low birth weight, and neonatal mortality. Evidence from coastal Bangladesh linked saline drinking water and elevated sodium intake during pregnancy to gestational hypertension and pre-eclampsia.^69^ Studies from India and Argentina further associated high temperatures with reduced sperm quality, concentration, motility, and morphology.^66^^,^^67^

Also featured in our typology of mechanisms based on the literature (Figure 4) are pre-existing contextual realities (i.e., those that predate the climate change exposure) grouped into 6 domains: (1) patriarchal norms, (2) cultural power structures, (3) socioeconomic realities, (4) gender inequitable climate governance, (5) place-based factors, and (6) pre-existing systems/infrastructure. These contextual factors can amplify the harmful impacts of climate change on women and girls compared to men and boys and disproportionately affect those at intersecting axes of marginalization, such as poverty and ethnicity.

Patriarchal norms place men in positions of power by limiting women’s and girls’ movement, decision-making, and bodily control as well as promoting harmful gender roles and the shame and stigma around women’s/girls’ bodies and sexualities. Women’s/girls’ limited decision-making and harmful gender norms concerning domestic labor were studied in about half of the pathways. For example, in situations of forced displacement, women and girls have less control over whether, where, and when to migrate compared to men. In crowded emergency camps, menstruation-related stigma marginalizes adolescent girls; pre-existing practices of sexual harassment (eve teasing) creates environments where girl child marriage is perceived as protective against GBV and a means to protect family honor. In Bangladesh, adolescent girls described feelings of shame and difficulty managing menstruation during flood-induced forced displacement due to gendered expectations around privacy and bodily cleanliness.^61^ Studies from Kenya,^39^ Tanzania,^38^ and Chad^98^ showed how drought-related water insecurity, male outmigration, and resource scarcity intensified domestic burdens for women and girls (reflecting the presence of gender inequitable norms).

Cultural power structures concern the ideologies that justify discrimination, such as colonialism, as well as context-specific forms of discrimination, such as the caste system. Dowry practices appeared in pathways investigating child marriage and GBV. Discrimination by caste or Indigenous identity were apparent relative to maternal-infant outcomes given discrimination-related differential access to care, social stigmatization, and the inequitable patterning of the social determinants of health.

Socioeconomic realities include existing gradients determined by poverty/debt, family structure and size, education level, and reliance on subsistence farming or agricultural work. Pre-existing gradients (related to income, education, and employment) and reliance on subsistence farming/agricultural work were most dominant, each studied by half of the pathways. For example, rural households relying on subsistence farming or fishing are among the most vulnerable to climate-related exposures, lacking economic and social protections for women and girls and persons living with HIV/AIDS. Further, pre-existing socioeconomic gradients moderated the risk of child marriage following drought. For example, evidence from Bangladesh noted that weather shocks (defined as dry months with precipitation significantly below the long-term average) had a larger (and more statistically significant) relationship with child marriage among the poorest upazilas compared to the less-poor upazilas.^94^ Rural communities that rely on subsistence farming or cash crops like cane sugar are particularly vulnerable to the socioeconomic consequences of drought. In Nepal, socially disadvantaged women faced heightened vulnerability due to chronic stressors that amplified the biological response to heat, including an acute cortisol surge entering placental circulation.^159^ In Pakistan, maternal nutritional supplementation prior to pregnancy emerged as a potential protective factor, attenuating the impact of heat on neonatal birth length.^142^

Gender-inequitable climate governance concerns gender-unequal and blind climate governance of warning systems, disaster preparedness, aid distribution, and water access. Warning and preparedness systems were deemed gender inequitable in some of the literature reviewed because women’s and girls’ lived realities were not considered in their development. For example, this was evident in limited consultations with women and girls, insufficient targeting of communication to reach them, and inadequate dissemination of information through their social and kinship networks. The mechanism linking climate disasters and salinity to menstrual health and hygiene was shaped by multiple gender-inequitable climate policies, including those related to warning systems, aid distribution, and water governance.^79^ The presence of early disaster warning systems and community-based educational opportunities have the potential to disrupt the harmful impacts of climate disasters on child marriage. Evidence emerged from an Indonesian study, which analyzed various contextual factors: availability of early warning systems, welfare status of villages, education facilities, and infrastructure.^114^ Early disaster warning systems reduced the probability of child marriage by 2.9 percentage points (*p* < 0.05).^114^ Secondly, the availability of community-based secondary school and skills training institutions reduced the probability of a girl entering child marriage.^114^ Each additional facility reduced the probability of a girl to be a child bride by 1.3% (secondary school) and 0.46% (skills training institutions).^114^

Place-based modifiers of climate change effects on SRHR reported in the reviewed literature included densely populated settlements and geographical realities like rural areas, living near or on riverbanks/coastal settlements, and lowland and island housing. For example, island communities in Bangladesh were also far away from healthcare facilities and relied on boats for transport, even prior to the onset of a climate disaster.^82^ The place-based factor reported most was living in rural areas, represented by half of the pathways investigated, relating to the differential effects of climate change on gestational hypertension, maternal-infant outcomes, fertility, and HIV/AIDS in rural compared to urban areas.

Contextual moderators in the category of pre-existing systems and infrastructure concerned the degree of resilience in water, sanitation, and hygiene (WASH); healthcare; transportation; energy; and education systems, as well as in the labor force. Weak medical infrastructure and water and sanitation systems as well as women’s/girls’ pre-existing education levels and employment access were each represented by about one-third of the pathways studied. Where systems and infrastructure are not resilient in the face of climate change exposures, there is greater risk for gender-inequitable SRHR outcomes. For example, an unreliable energy grid increases the risk of birth complications and magnifies risk of opportunistic violence against displaced women and girls in emergency shelters. When schools cannot resume their function following an EWE, adolescent girls are deprived of educational opportunities that delay marriage and childbearing.

## Discussion

This scoping review synthesized evidence from 256 empirical studies exploring the relationship between climate change and SRHR in LMICs. The LMIC-focused literature is concentrated on quantitative research conducted in South and East Asia (mainly China and Bangladesh) and Sub-Saharan Africa. Most evidence focused on temperature, floods, and droughts. Maternal infant health outcomes emerged as the most frequently studied SRHR domain. In contrast, domains such as abortion, psychosexual health, and SRHR education were rarely addressed. We synthesized 14 possible pathways linking climate-related exposures to SRHR outcomes. The pathways are underpinned by a typology of mediators and contextual moderators, which could advance a mechanistic understanding of climate change impacts on SRHR.

Just as climate impacts are shaped by context, the evidence available to understand the relationship between climate change and SRHR is also shaped by geopolitical and funding contexts. Certain climate exposures and SRHR outcomes dominate not only because they are important but also because research funding priorities shape what gets studied. The concentration of funding organizations in a relatively small number of countries highlights that knowledge production on climate change and SRHR is itself embedded within global funding structures. These patterns may influence which research questions are prioritized, which populations are studied, and which pathways receive empirical attention. Consequently, the gaps identified in this review may reflect not only evidence deficits but also broader inequities in research investment and agenda setting.

In 2023, a Delphi consultation established expert-identified research priorities across on climate change and SRHR in LMICs and high-income countries.^160^ This initiative called for investments in better SRHR indicators, intersectional analytical frameworks, and rigorous methodologies, particularly longitudinal studies and impact evaluations to map the casual pathways between climate change and SRHR outcomes.^160^ Funders can additionally support research that tests and refines our understanding of the mechanisms underlying harmful associations for the purpose of designing relevant adaptation responses.

Investigating theoretical causal pathways between climate change and SRHR enables researchers and practitioners to more deeply understand underlying processes of harm and strategic intervention points in a contextually relevant manner. Accordingly, our work contributes to the development of more effective, gender-responsive climate action and adaptation plans that fully incorporate SRHR dimensions.

The findings from our review could advance intervention and policy design in several ways: (1) contextualization of harmful associations (harmful effects are not equally harmful across populations and regions), (2) target improvement (by strategically identifying and acting on mediators that induce harm), and (3) revealing important heterogeneity regarding opportunities for protection based on contextual features. As our synthesis demonstrates, analysis of mediators helps explain how or why climate change yields deleterious SRHR outcomes and analysis of contextual moderations helps explain under what conditions and for whom effects are most harmful.

Future research can apply causal thinking to more deeply explore the mechanisms between climate change and SRHR in distinct LMICs, making broader connections to other aspects of quality of life. For example, attention should be given to investigating the possible mediating paths identified in this review as well as effect moderation and statistical interaction based on contextual moderators. Understanding these mechanisms is essential for identifying how climate change exacerbates downstream SRHR outcomes and designing interventions that prevent and mitigate harmful pathways.^160^ Moreover, additional research is needed to further distinguish pathways of harm involving extreme events and gradual changes in climate indicators. In our review, chronic temperature changes were related to biological mechanisms that affected birth outcomes like pregnancy loss and anthropometry and fertility. Evidence for a more diverse set of SRHR outcomes was available with respect to droughts and EWEs like floods and storms. Lastly, adopting a polycrisis lens can clarify the interrelated relationship between climate change and other macro-level stressors in LMICs, like conflict.^161^^,^^162^ Though conflict did not emerge as a contextual or mediating construct in the studies analyzed, its patterning in climate-affected countries warrants further research.

Further, interventions should be developed in discussion and dialog with diverse community members and frontline workers at the field level in LMICs, reflecting the lived experiences of those most affected.^160^^,^^163^ In terms of intervention adaptation, implementation science can prioritize both contextual moderators and pathway mediators, while centering localized knowledge to effectively embed SRHR into climate adaptation strategies.^164^ Investing in social science research in these areas can support the development of more contextually relevant, equitable, climate-resilient health systems that protect and promote SRHR in the face of ongoing climate change disasters.

In our review, climate change disasters and alterations in climate indicators engendered infrastructure damage, threatened livelihoods, and intensified stress and lost social support due to migration and mortality. Pre-existing and contextual gendered power structures that reduced women’s decision-making, autonomy, and degree of household/parenting labor also intensified. Thus, in the absence of gender protections, informed by a mechanistic understanding of harmful pathways, opportunities to advance the human capital of women, girls, and gender minorities dwindle as climate change continues to unfold. These consequences are most pronounced in communities where women and girls are already marginalized and unable to experience their full human rights.

As climate shocks intensify, so do existing structural inequalities: discriminatory norms, restricted access to reproductive care, and intensified domestic labor.^165^ These barriers heighten vulnerabilities, disrupt education and SRHR services, and threaten fundamental rights and bodily autonomy.^166^ Yet climate governance remains overwhelmingly gender blind, meaning it does not consider the existing gender norms and social relations, reinforces gender-based discrimination, and ignores gender differences in resource allocation.^167^

Given both the intensification of climate change and gender equality backsliding, policies and future research must move toward transformative change.^168^^,^^169^ There are several ways to promote transformation, informed by causal thinking. For example, in our synthesis, floods, storms, and drought adversely affected men’s traditional gender role of being an economic provider, primarily among households relying on agriculture and fishing in rural areas. There is an opportunity to prevent incident and lethal cases of violence against women under these conditions by focusing on men’s mental/emotional well-being and maladaptive coping via substance use and by providing social protection programs to female household members who are above a certain age (to disincentivize child marriage as a mechanism for creating more households).

Future research should be locally grounded and gender focused. Funding should prioritize participatory research models that center the experiences of key groups.^170^ Adolescence is a sensitive period of physical, emotional, and social development for girls and presents a window for policy action. For example, over 880 million adolescent girls and young women globally face menstruation within the wider context of food insecurity, early marriage and early childbearing, and sexual violence, all of which are intensified by climate shocks.^171^ In Kenya, girls are pushed into exploitative relationships during droughts to access basic needs like menstrual supplies, yet menstrual health under climate stress remains poorly understood.^22^ In Northern Ghana, for instance, women reported using rags, leaves, or tissues during menstruation due to climate-induced economic strain, leading to adverse health effects.^170^ Similarly, in flood-prone regions of Bangladesh, women reported prolonged use of hormonal contraceptives to avoid menstruation, as they lacked access to WASH infrastructure.^170^ These examples illustrate how climate change directly affects menstrual health: a subject that remains critically under-researched in general and from a mechanistic perspective.^172^

This review has several limitations. First, although studies represented 108 LMICs, the evidence base was geographically concentrated in a relatively small number of countries, particularly China, Bangladesh, India, Uganda, Pakistan, Malawi, and Kenya, which may limit the transferability of findings to less-studied settings. Second, the literature was heavily concentrated on maternal-infant outcomes, temperature, floods, and droughts, while comparatively little evidence was available for abortion, psychosexual health, and menstrual health and hygiene, and no studies examined the outcomes related to comprehensive sexuality education and information. This uneven distribution of evidence influenced the pathways and mechanisms that could be identified. Third, while our review sought to advance causal thinking by synthesizing reported mediators and contextual moderators, most included studies were observational, cross-sectional, or qualitative in design, limiting the ability to draw causal conclusions regarding the pathways identified. Further, the pathways presented in this review should not be interpreted as universally established causal mechanisms. Although some included studies investigated mechanisms, many proposed pathways were derived from qualitative evidence describing lived experiences or from synthesis-level integration across studies of varying study designs. Accordingly, the framework is best understood as identifying plausible and evidence-informed mechanisms requiring further empirical testing rather than confirming causal pathways. Fourth, the review was restricted to English-language, peer-reviewed publications and excluded gray literature; therefore, relevant evidence from non-English sources, government reports, technical documents, and programmatic evaluations may not have been captured. Consistent with scoping review methodology, we did not conduct a formal quality appraisal or risk-of-bias assessment, and the findings should, therefore, be interpreted as a mapping of the available evidence rather than an assessment of evidence certainty. Finally, publication bias may have affected the available evidence base, as studies reporting significant or novel findings are more likely to be published than studies reporting null or inconsistent results. Together, these limitations underscore the need for more geographically diverse, methodologically rigorous, and under-represented areas of SRHR research within the climate change literature.

## Conclusions

Overall, our work underscores the need to integrate SRHR into climate change discourse, policy, and action, particularly in LMICs. Mechanistic thinking offers a powerful lens to move beyond correlation and toward a causal understanding of how and why SRHR inequities persist and are magnified by climate change. As climate shocks intensify and global funding landscapes shift, it is imperative that SRHR is not sidelined. Future research can deepen causal inquiry, expand geographic and thematic coverage, and elevate under-represented voices with respect to designing and testing interventions.

### Data sharing

All extracted data and information are available at https://doi.org/10.6084/m9.figshare.33489616.

## Acknowledgments

This work was carried out with the aid of a grant from the 10.13039/501100000193International Development Research Centre, Ottawa, Canada (grant no. 110623-001). The funders had no role in the design, analysis, interpretation, writing, or decision to submit this manuscript. The views expressed herein do not necessarily represent those of IDRC or its Board of Governors. We also acknowledge the supportive efforts of two summer interns: Ms. Julia Smolik and Ms. Julia Tikhonov.

## Author contributions

L.V., conceptualization, data curation, formal analysis, funding acquisition, investigation, methodology, project administration, resources, software, supervision, validation, visualization, writing – original draft, and writing – review and editing; S.P., formal analysis, investigation, validation, visualization, writing – original draft, and writing; S.Z., formal analysis, investigation, validation, visualization, writing – original draft, and writing; V.B., validation, investigation, and writing – review and editing; S.F.R., validation, investigation, and writing – review and editing; M.E.A., validation, investigation, and writing – review and editing; R.H., validation, investigation, writing – review and editing; M.G., validation, investigation, writing – review and editing, and funding acquisition; Z.A.B., validation, investigation, writing – review and editing, and funding acquisition.

## Declaration of interests

The authors declare no competing interests.

## Supplemental information

Document S1. Tables S1–S3 and Texts S1 and S2
