# Supplementary material for: Climate change and sexual and reproductive health: A mixed methods scoping review of possible mechanisms
Source: iScience. 2026 Oct 12;29(11):117794. doi: 10.1016/j.isci.2026.117794 (PMC13682385; doi:10.1016/j.isci.2026.117794)
Supplement: Supplementary file 1 [file mmc1.pdf]

iScience, Volume *29*

Supplemental information

Climate change and sexual and reproductive health: A mixed methods scoping review of possible mechanisms

Luissa Vahedi, Susan Pradhan, Sakina Zaidi, Vanessa Brizuela, Sabina Faiz Rashid, Maha El Adawy, Rana Hajjeh, Michelle Gaffey, and Zulfiqar A. Bhutta

Supplemental Information (SI)

Contents

Supplemental Information (SI) 1
- Table S1. Embase search strategy 2
- Table S2. Medline search strategy 18
- Text S1: Scopus search strategy 32
- Table S3. Web of science search strategy 36
- Text S2: Data extraction/Information charting 45

Table S1. Embase search strategy

1.	south asian/ or afghan/ or bangladeshi/ or bhutanese/ or indian/ or nepalese/ or pakistani/ or sri lankan/
2.	*southeast asia/ or indonesia/ or thailand/ or timor-leste/
3.	iran/ or iraq/ or jordan/ or kuwait/ or lebanon/ or oman/ or palestine/ or qatar/ or saudi arabia/ or syrian arab republic/ or united arab emirates/ or yemen/
4.	Myanmar/
5.	bahrain/ or western asia/
6.	exp North Korea/
7.	Timor-Leste/
8.	south asia/ or afghanistan/ or bangladesh/ or bhutan/ or india/ or maldives/ or nepal/ or pakistan/ or sri lanka/
9.	southeast asian/ or burmese/ or cambodian/ or indonesian/ or "thai (citizen)"/
10.	west asian/ or emirati/ or "iranian (citizen)"/ or iraqi/ or jordanian/ or kuwaiti/ or lebanese/ or omani/ or palestinian/ or qatari/ or saudi/ or syrian/ or yemeni/
11.	("Middle East*" or "South* Asia*" or "Southeast Asia*" or "South east Asia*" or "Eastern Mediterranean Region*" or "South-East Asia Region*").mp. [mp=title, abstract, heading word, drug trade name, original title, device manufacturer, drug manufacturer, device trade name, keyword heading word, floating subheading word, candidate term word]
12.	(Iran or Bahrain or Iraq or Jordan or Kuwait or Lebanon or Oman or Qatar or "Saudi Arabia" or Syria or "United Arab Emirates" or Yemen or "Democratic People's Republic of Korea" or "North Korea" or Myanmar or Indonesia or Thailand or "Timor-leste" or Afghanistan or Bangladesh or Bhutan or India or Maldives or Nepal or Pakistan or "Sri Lanka" or Palestine or "West Bank" or Gaza or Iranian* or Bahraini* or Iraqi* or Jordanian* or Kuwaiti* or Lebanese or Omani* or Qatari* or "Emirati*" or Syrian* or "Saudi Arabian*" or saudi* or Yemeni* or "Democratic People's Republic of Korea*" or Burmese or Myanma or Indonesian* or Thai or Timorese or Afghani* or Bengali* or Bhutanese or Indian* or Maldivian* or

	Nepalese or Nepali* or Pakistani* or "Sri Lankan" or Palestinian* or "Gazan*").mp. [mp=title, abstract, heading word, drug trade name, original title, device manufacturer, drug manufacturer, device trade name, keyword heading word, floating subheading word, candidate term word]
13.	exp Bahraini/
14.	exp North Korean/
15.	1 or 2 or 3 or 4 or 5 or 6 or 7 or 8 or 9 or 10 or 11 or 12 or 13 or 14
16.	climate change/ or climate warming/
17.	"Climate change".mp. [mp=title, abstract, heading word, drug trade name, original title, device manufacturer, drug manufacturer, device trade name, keyword heading word, floating subheading word, candidate term word]
18.	("heavy rainfall*" or ((chang* or shift*) adj2 weather) or "weather pattern?" or "rising sea level?").mp. [mp=title, abstract, heading word, drug trade name, original title, device manufacturer, drug manufacturer, device trade name, keyword heading word, floating subheading word, candidate term word]
19.	((Glacier* or glacial* or iceberg* or "sea ice") adj2 (melt* or shrink* or sublimat*)).mp. [mp=title, abstract, heading word, drug trade name, original title, device manufacturer, drug manufacturer, device trade name, keyword heading word, floating subheading word, candidate term word]
20.	deglaciation/
21.	high temperature/
22.	extreme hot weather/ or heat wave/
23.	("Heat -related" or "hot weather" or "extreme heat" or "Extreme temperature?" or "Extreme Hot Weather" or "heat wave" or heatwave or "high temperature?" or "rising temperature?" or "extreme cold").mp. [mp=title, abstract, heading word, drug trade name, original title, device manufacturer, drug manufacturer, device trade name, keyword heading word, floating subheading word, candidate term word]

24.	*natural disaster/
25.	avalanche/
26.	exp hurricane/ or exp severe weather/
27.	drought/
28.	flooding/
29.	exp landslide/
30.	exp tornado/
31.	wildfire/ or forest fire/
32.	exp extreme weather/
33.	("severe weather" or "extreme weather" or Drought? or Flood* or "natural disaster?" or Hurricane* or Wildfire* or "forest fire" or wild-fire* or tornado* or "Tropical storm" or avalanche? or cyclone* or landslide? or "land slide" or mudslide? or "mud slide?" or storm? or tornado* or typhoon?).mp. [mp=title, abstract, heading word, drug trade name, original title, device manufacturer, drug manufacturer, device trade name, keyword heading word, floating subheading word, candidate term word]
34.	(((emergency or emergencies) adj5 (environ* or human or manmade or "man made" or nature or natural or weather or respons*)) or ("heavyrainfall*" or ((chang* or shift*) adj2 weather) or "weather pattern?" or "rising sea level?")).mp. [mp=title, abstract, heading word, drug trade name, original title, device manufacturer, drug manufacturer, device trade name, keyword heading word, floating subheading word, candidate term word]
35.	sea level rise/
36.	(("Climate change*" or "climate change-related" or "climate-related") adj2 (windspeed? or "wind speed?" or "global stilling" or precipitation* or humid*)).mp. [mp=title, abstract, heading word, drug trade name, original title, device

	manufacturer, drug manufacturer, device trade name, keyword heading word, floating subheading word, candidate term word]
37.	(("Climate change*" or "climate change-related" or "climate-related") and (distribut* or spread* or transmi* or vector?) and (pathogen* or "disease? or infectious disease?" or virus* or bacteria*)).mp. [mp=title, abstract, heading word, drug trade name, original title, device manufacturer, drug manufacturer, device trade name, keyword heading word, floating subheading word, candidate term word]
38.	(exp Climate Change/ or exp Extreme Weather/) and (communicable disease/ or blood-borne infection/ or water borne disease/ or insect vector/ or mosquito vector/)
39.	greenhouse effect/
40.	exp greenhouse gas/
41.	((("greenhouse effect" or GHG? or Greenhouse Gas* or "carbon dioxide" or "CO2" or "CO2 equivalent*" or "nitrous oxide" or CH4 or "Sulphurdioxide" or "Sulfur dioxide" or "SO2" or "NO2" or "combustion air pollution" or Chlorofluorocarbon* or trifluoride* or Methane or ammonia ornitrogen or CFC? or "nitrogen trifluoride*" or NF3 or Hydrofluorocarbon* or perfluorocarbon* or "sulfur hexafluoride" or "sulphur hexafluoride orSF6") adj2 (increas* or level? or Atmospher* or emission*)) or "global warming" or (environmental adj degrad*)).mp. [mp=title, abstract, heading word, drug trade name, original title, device manufacturer, drug manufacturer, device trade name, keyword heading word, floating subheading word, candidate term word]
42.	air pollution/ or ozone depletion/
43.	(climate change/ and air pollution/) or (("Climate change*" or "climate change-related" or "climate-related") and (air adj2 (quality or pollut*))).mp. [mp=title, abstract, heading word, drug trade name, original title, device manufacturer, drug manufacturer, device trade name, keyword heading word, floating subheading word, candidate term word]
44.	water quality/
45.	water quality/ or ((water or groundwater) adj (ground or quality or potab* or safe* or drink* or suppl* or secur* or insecur*)).mp. or ((Saltwater or " salt water" or "sea water") adj2 encroach*).mp. or (Salinat* adj2 (water or soil)).mp. [mp=title,

	abstract, heading word, drug trade name, original title, device manufacturer, drug manufacturer, device trade name, keyword heading word, floating subheading word, candidate term word]
46.	((climat* or "climate-related" or "climate change-related" or environment* or "environmentally-induced") adj2 ("human security" or change or warming or emergenc* or hazard* or cris?s or refugee* or shock* or variabilit* or displac* or "forced migration" or (availab* adj3 (food* or water or food system*)) or agricultur* or famine* or war* or armed conflict* or political conflict* or violen*)).mp. [mp=title, abstract, heading word, drug trade name, original title, device manufacturer, drug manufacturer, device trade name, keyword heading word, floating subheading word, candidate term word]
47.	(exp Climate Change/ or exp Extreme Weather/) and (exp refugee camp/ or exp refugee/ or exp refugee crisis/ or exp agriculture/ or exp food security/ or exp hunger/ or exp crop production/ or exp agricultural land/ or exp water supply/)
48.	16 or 17 or 18 or 19 or 20 or 21 or 22 or 23 or 24 or 25 or 26 or 27 or 28 or 29 or 30 or 31 or 32 or 33 or 34 or 35 or 36 or 37 or 38 or 39 or 40 or 41 or 42 or 43 or 44 or 45 or 46 or 47
49.	(human/ and (exp precocious puberty/ or exp delayed puberty/ or exp puberty/ or exp sexual maturation/)) or (menarche* or "menstrual cycle" or menstruation).mp. [mp=title, abstract, heading word, drug trade name, original title, device manufacturer, drug manufacturer, device trade name, keyword heading word, floating subheading word, candidate term word]
50.	exp menarche/
51.	sexual education/
52.	exp reproductive health/ or exp sexual education/
53.	("family planning education" or "family planning instructor*" or "family planning training" or "contraceptive counselling" or "family planning programs" or "sex education" or "sex instruction" or "sexual health" or "menstrual hygiene").mp.
54.	exp infertility/ or exp female infertility/ or exp male infertility/ or exp infertility therapy/

55.	("Reproductive Sterility" or sterility or Subfertility or Sub-Fertility or "female infertility" or "male infertility" or infertil*).mp. [mp=title, abstract, heading word, drug trade name, original title, device manufacturer, drug manufacturer, device trade name, keyword heading word, floating subheading word, candidate term word]
56.	contraception/ or birth control/ or barrier contraception/ or emergency contraception/ or hormonal contraception/ or long-acting reversible contraception/ or oral contraception/ or ovulation inhibition/ or reproductive sterilization/ or vagina contraception/
57.	family planning/ or birth interval/ or pregnancy interval/
58.	coitus interruptus/ or contraceptive behavior/ or penetrative sex/
59.	postcoitus contraceptive agent/ or contraceptive agent/
60.	exp barrier contraception/
61.	exp emergency contraception/
62.	condom/ or male contraceptive device/
63.	exp contraceptive device/
64.	sexual dysfunction/ or female sexual dysfunction/ or male sexual dysfunction/ or orgasm disorder/ or sexual arousal disorder/ or sexual desire disorder/ or sexual pain/
65.	("sexual dysfunction" or "female sexual dysfunction" or "male sexual dysfunction" or "orgasm disorder*" or "sexual arousal disorder*" or "sexual desire disorder*" or "sexual pain").mp. [mp=title, abstract, heading word, drug trade name, original title, device manufacturer, drug manufacturer, device trade name, keyword heading word, floating subheading word, candidate term word]
66.	psychosexual disorder/ or sexual desire disorder/ or sexual dissatisfaction/ or vaginism/
67.	("psychosexual disorder*" or "sexual desire disorder*" or "sexual dissatisfaction" or "vaginism").mp. [mp=title, abstract, heading word, drug trade name, original title,

	device manufacturer, drug manufacturer, device trade name, keyword heading word, floating subheading word, candidate term word]
68.	("birth regulation" or "birth control*" or "coitus interruptus" or "fertility control" or contracept* or "unprotected sex" or "unsafe sex" or antifertility or anticonception or "fertility control" or "fertilization inhibition" or "inhibition of fertilization" or "fertilisation inhibition" or "inhibition of fertilisation" or "morning after pill" or "postcoital antifertility agent" or "postcoital pill").mp. [mp=title, abstract, heading word, drug trade name, original title, device manufacturer, drug manufacturer, device trade name, keyword heading word, floating subheading word, candidate term word]
69.	("cervical cap" or "cervical caps" or "cervix cap" or "dalkon shield" or "dana deveice" or "i.u.d." or "intra uterine device*" or "intracervical device" or "intrauterine device*" or "intrauterine devices" or "intravaginal pessaries" or "intravaginal pessary" or "iucd" or "iud" or "iuds" or "Lippes loop" or "marguilies spiral" or "margulie coil" or "progestin implant" or "vagina pessaries" or "vagina pessary" or "vaginal diaphragm" or "vaginaldiaphragms" or "vaginal pessaries" or "vaginal pessary" or "vaginal ring" or "vaginal shield" or "vaginal rings" or "vaginal shields" or "vaginalsponge" or "vaginal sponges" or condom or condom*).mp. [mp=title, abstract, heading word, drug trade name, original title, device manufacturer, drug manufacturer, device trade name, keyword heading word, floating subheading word, candidate term word]
70.	exp induced abortion/ or exp septic abortion/ or exp therapeutic abortion/ or exp medical abortion/ or exp illegal abortion/ or exp abortion/ or exp spontaneous abortion/ or exp surgical abortion/ or exp legal abortion/
71.	(abortion* or abortus).mp. or Misoprostol/ or Mifepristone/ [mp=title, abstract, heading word, drug trade name, original title, device manufacturer, drug manufacturer, device trade name, keyword heading word, floating subheading word, candidate term word]
72.	exp mifepristone plus misoprostol/
73.	("birth interval" or "birth intervals" or "birth spacing" or "birth spacings" or "child spacing" or "child spacings" or "family building" or "family planning" or "pregnancy interval" or "pregnancy intervals").mp. [mp=title, abstract, heading word, drug trade

	name, original title, device manufacturer, drug manufacturer, device trade name, keyword heading word, floating subheading word, candidate term word]
74.	exp gynecology/ or exp obstetrics/ or *pregnancy/
75.	(gynaecology or gynecology or "reproductive health" or "sexual health").mp. [mp=title, abstract, heading word, drug trade name, original title, device manufacturer, drug manufacturer, device trade name, keyword heading word, floating subheading word, candidate term word]
76.	maternal welfare/ or maternal care/
77.	("maternal health maternal welfare" or "maternal child health" or "maternal child welfare" or obstetric* or "minimum initial service package" or "maternal welfare").mp.
78.	exp maternal mortality/
79.	exp midwife/
80.	("birth attendant" or "birth attendants" or midwife or midwives or midwifery).mp.
81.	(postnatal or "post natal" or postpartum or "post partum" or puerperium or puerperal).mp.
82.	postnatal care/ or puerperium/
83.	exp postpartum hemorrhage/
84.	birth/
85.	("safe delivery" or "safely delivered" or (antepartum or "ante partum" or intrapartum or "intra partum")).mp. [mp=title, abstract, heading word, drug trade name, original title, device manufacturer, drug manufacturer, device trade name, keyword heading word, floating subheading word, candidate term word]
86.	("prenatal" or "pre natal" or "antenatal" or "ante natal").mp. [mp=title, abstract, heading word, drug trade name, original title, device manufacturer, drug

	manufacturer, device trade name, keyword heading word, floating subheading word, candidate term word]
87.	("perinatal care" or "peri natal care" or "perinatal care" or "peri natal care").mp. [mp=title, abstract, heading word, drug trade name, original title, device manufacturer, drug manufacturer, device trade name, keyword heading word, floating subheading word, candidate term word]
88.	("peripartum* period* or perinatal* period*" or "peri natal* period*").mp. [mp=title, abstract, heading word, drug trade name, original title, device manufacturer, drug manufacturer, device trade name, keyword heading word, floating subheading word, candidate term word]
89.	stillbirth/ or fetus death/
90.	(stillbirth* or stillborn* or "still birth*" or "still born*" or "still born*" or miscarriage*).mp. [mp=title, abstract, heading word, drug trade name, original title, device manufacturer, drug manufacturer, device trade name, keyword heading word, floating subheading word, candidate term word]
91.	("abnormal labor" or "delayed labor" or "delayed labour" or "inertia uteri" or "labor obstruction" or "labour obstruction" or "obstructed labor" or "obstructed labour" or "uterus inertia" or dystocia or dystocias).mp. [mp=title, abstract, heading word, drug trade name, original title, device manufacturer, drug manufacturer, device trade name, keyword heading word, floating subheading word, candidate term word]
92.	(breech adj2 (present* or position*)).mp. [mp=title, abstract, heading word, drug trade name, original title, device manufacturer, drug manufacturer, device trade name, keyword heading word, floating subheading word, candidate term word]
93.	("vagina* haemorrhage" or "vagina* hemorrhage" or "vaginal bleeding").mp. [mp=title, abstract, heading word, drug trade name, original title, device manufacturer, drug manufacturer, device trade name, keyword heading word, floating subheading word, candidate term word]
94.	breech presentation/
95.	exp uterus bleeding/

96.	((discriminat* or stigma* or "social exclusion*" or persecut* or violen*) adj3 (Non-Heterosexual? or GLBT or LGBTQ or "Gender Divers*" or LBG or LGBT or "Sexual Minorit*" or LGBTQIA or Bisexual* or lesbian* or gay* or homosexual* or Queer* or "Men Who Have Sex With Men" or MSM or "Women Who Have Sex With Women" or WSW or transgender* or transsexual* or trans-gender* or Transmen or transman or trans-sexual* or intersex* or inter-sex* or 2spirit* or twospirit* or "2 Spirit*" or "two spirit*" or trans-women or trans-woman or "Gender Minorit*")).mp. [mp=title, abstract, heading word, drug trade name, original title, device manufacturer, drug manufacturer, device trade name, keyword heading word, floating subheading word, candidate term word]
97.	("Social Discrimination"/ or "Social Stigma"/) and ("Sexual and Gender Minorities"/ or "Gender Identity"/ or Transsexualism/ or transgenderism/)
98.	exp gender equity/ or exp women's rights/
99.	sex worker/
100.	(gender adj3 (equalit* or equity or identit* or norm? or role or violence)).mp. [mp=title, abstract, heading word, drug trade name, original title, device manufacturer, drug manufacturer, device trade name, keyword heading word, floating subheading word, candidate term word]
101.	(women* adj2 (liberation or right* or status)).mp. [mp=title, abstract, heading word, drug trade name, original title, device manufacturer, drug manufacturer, device trade name, keyword heading word, floating subheading word, candidate term word]
102.	*Human immunodeficiency virus infection/
103.	*acquired immune deficiency syndrome/
104.	("aids" or "acquired immune deficiency syndrome*" or "acquired immuno deficiency syndrome* acquired immunodeficiency syndrome*").mp. [mp=title, abstract, heading word, drug trade name, original title, device manufacturer, drug manufacturer, device trade name, keyword heading word, floating subheading word, candidate term word]
105.	vertical transmission/

106. (pmtct or "prevention of HIV mother to child transmission" or "prevention of mother to child transmission" or "eliminate mother to child transmission" or "prevention of mother to child HIV transmission").mp. [mp=title, abstract, heading word, drug trade name, original title, device manufacturer, drug manufacturer, device trade name, keyword heading word, floating subheading word, candidate term word]

107. ("human immunodeficiency virus" or HIV).mp. [mp=title, abstract, heading word, drug trade name, original title, device manufacturer, drug manufacturer, device trade name, keyword heading word, floating subheading word, candidate term word]

108. exp sexually transmitted disease/

109. ("sexually transmitted disease*" or "sexually transmitted infection*" or "venereal disease*" or "venereal infection" or "venereal infections").mp. [mp=title, abstract, heading word, drug trade name, original title, device manufacturer, drug manufacturer, device trade name, keyword heading word, floating subheading word, candidate term word]

110. ("sexually transmitted" or STI* or STD*).mp.

111. sexually transmitted disease/ or bacterial sexually transmitted disease/ or viral sexually transmitted disease/

112. exp ulcus molle/

113. Chlamydia infection/

114. exp lymphogranuloma venereum/

115. gonorrhea/

116. exp syphilis/

117. exp genital herpes/

118. exp condyloma acuminatum/

119. ("calymmatobacterium granulomatis infection" or "chancroid" or "chancroids" or chlamidiosis or "chlamydia infection" or "chlamydia infections" or "Donovania

	granulomatis infection" or "donovanosis" or "frei disease" or "gonococcal infection" or "gonococcal infections" or "gonococcosis" or "gonococcus or infection" or "gonorrhea" or "gonorrheas" or "gonorrhoea" or "granuloma inguinale" or "granuloma or venereum" or "great pox" or "inguinal lymphogranulomatosis" or "Klebsiella granulomatis infection" or "lues" or "lymphogranuloma inguinale" or "lymphogranuloma venereum" or "lymphogranuloma venerum" or "lymphopathia venerea" or "lymphopathia venereum" or "Neisseria gonorrhoeae infection" or "nicholas favre disease" or "nicolas favre disease" or "syphilis" or "syphilitic disorder" or "venereal lymphogranuloma" or "HPV" or "human papilloma virus" or "human papillomavirus").mp. [mp=title, abstract, heading word, drug trade name, original title, device manufacturer, drug manufacturer, device trade name, keyword heading word, floating subheading word, candidate term word]
120.	("anal wart" or "anal warts" or "anogenital wart" or "anogenital warts" or "condyla acuminatum" or "condylatum acuminatum" or "condyloma accuminatum" or "condyloma acuminata" or "condylomata acuminata" or "genital herpes" or "genital wart" or "genital warts" or "herpes genitalis" or "herpes or progenitalis" or "herpes simplex genitalis" or "herpes simplex virus genital infection" or "penile wart" or "penile warts" or "perianal wart" or "perianal warts" or "venereal wart" or "venereal warts" or "verruca or accuminata" or "vulvar condyloma").mp. or Scabies/ or ("sarcoptic mange" or scabies).mp. [mp=title, abstract, heading word, drug trade name, original title, device manufacturer, drug manufacturer, device trade name, keyword heading word, floating subheading word, candidate term word]
121.	exp Phthirus/
122.	("crab lice" or "crab lices" or "crab louse" or "crab louses" or "Pediculus pubis" or "phthirus" or "Phtiruspubis" or "Pthirus pubis" or "pubic lice" or "pubic louse").mp. [mp=title, abstract, heading word, drug trade name, original title, device manufacturer, drug manufacturer, device trade name, keyword heading word, floating subheading word, candidate term word]
123.	partner violence/ or domestic violence/ or marital rape/
124.	gender based violence/ or femicide/
125.	exp sexual violence/ or exp rape/ or exp sexual abuse/ or exp sexual crime/
126.	exp emotional abuse/

127.	exp physical abuse/
128.	exp physical violence/
129.	exp emotional abuse/
130.	exp forced marriage/
131.	female genital mutilation/
132.	exp honor-based violence/
133.	exp battered woman/
134.	exp fistula/ or exp rectovaginal fistula/
135.	("partner abuse" or "partner violence" or "wife abuse" or "spouse abuse" or "spousal abuse" or "domestic violence" or "domestic abuse" or "gender based violence" or GBV or "violence against women and girls" or "sex* based violence" or "Dowry Death?" or hono?r killing* or "Hono?r Based Violence" or "Hono?r Crime*" or ((abuse* or assault* or violence) adj2 (woman or women)) or ("coerced intercourse" or "forced prostitution" or "forced sex" or "human trafficking" or "human traffickings" or rape* or "sex trafficking" or "sex traffickings" or "sex* abuse*" or "sex* assault*" or "sex* crime*" or "sex* offense" or "sex* offenses" or "sex* slave*" or "sexual aggression" or "sexual bullying" or "sexual coercion" or "sexual exploitation*" or "sexual harassment" or "sexual trauma" or "sexual violence" or "emotional violence" or IPV or FGM or "intimate partner violence" or "female genital mutilation" or female circumcision or Clitoridectom* or Clitorectom* or Infibulation? or "genital cutting" or "child marriage" or femicide or fillicide) or ("physical* abuse*" or "physical* assault*" or "physical violence")).mp. [mp=title, abstract, heading word, drug trade name, original title, device manufacturer, drug manufacturer, device trade name, keyword heading word, floating subheading word, candidate term word]
136.	human trafficking/ or sex trafficking/
137.	(fistula or fistulas or "genital trauma" or "genital injury" or "vaginaltrauma" or "vaginal injury").mp.

138.	exp female genital mutilation type III/
139.	((child or early or forced) adj marriage).mp. [mp=title, abstract, heading word, drug trade name, original title, device manufacturer, drug manufacturer, device trade name, keyword heading word, floating subheading word, candidate term word]
140.	low birth weight/ or small for gestational age/ or very low birth weight/
141.	("low birth weight" or "small for gestational age").mp. [mp=title, abstract, heading word, drug trade name, original title, device manufacturer, drug manufacturer, device trade name, keyword heading word, floating subheading word, candidate term word]
142.	49 or 50 or 51 or 52 or 53 or 54 or 55 or 56 or 57 or 58 or 59 or 60 or 61 or 62 or 63 or 64 or 65 or 66 or 67 or 68 or 69 or 70 or 71 or 72 or 73 or 74 or 75 or 76 or 77 or 78 or 79 or 80 or 81 or 82 or 83 or 84 or 85 or 86 or 87 or 88 or 89 or 90 or 91 or 92 or 93 or 94 or 95 or 96 or 97 or 98 or 99 or 100 or 101 or 102 or 103 or 104 or 105 or 106 or 107 or 108 or 109 or 110 or 111 or 112 or 113 or 114 or 115 or 116 or 117 or 118 or 119 or 120 or 121 or 122 or 123 or 124 or 125 or 126 or 127 or 128 or 129 or 130 or 131 or 132 or 133 or 134 or 135 or 136 or 137 or 138 or 139 or 140 or 141
143.	(afghan* or africa* or albania* or algeria* or angola* or antigua* or barbuda* or argentin* or armenia* or aruba* or azerbaijan* or bahrain* or bangladesh* or bengal* or bangal* or barbados* or barbadian* or bajan or bajans or belarus* or belorus* or byelarus* or byelorus* or belize* or benin* or dahomey or bhutan* or bolivia* or bosnia* or herzegovin* or botswan* or batswan* or bechuanaland* or brazil* or brasil* or bulgaria* or burkina* or burkinese* or upper volta* or burundi* or urundi* or cabo verde* or cape verde* or cambodia* or kampuchea* or khmer* or cameroon* or cameroun* or ubangi shari* or chad* or chile* or china* or chinese or colombia* or comoro* or comore* or comorian* or mayotte* or congo* or zaire* or costa rica* or "cote d'ivoir*" or "cote d' ivoir*" or cote divoir* or cote d ivoir* or ivory coast* or ivorian* or croatia* or cuba or cuban or cubans or "cuba's" or cyprus* or cypriot* or czech* or djibouti* or french somaliland* or dominica* or ecuador* or egypt* or united arab republic* or el salvador* or salvadoran* or guinea* or equatoguinea* or eritrea* or estonia* or eswatini* or swaziland* or swazi* or swati* or ethiopia* or fiji* or gabon* or gabonese* or gabonaise* or gambia* or ((georgia or georgian or georgians) not (atlanta or california or florida)) or ghana* or gibraltar* or greece* or greek* or grecian* or grenada* or grenadian* or guam* or guatemala* or guyana* or guiana* or guyanese* or haiti* or hispaniola* or hondura*

or hungary* or hungarian* or india* or indonesia* or iran* or iraq* or isle of man* or jamaica* or jordan* or kazakh* or kenya* or karabati* or korea* or kosovo* or kosova* or kyrgyz* or kirgiz* or kirghiz* or laos or lao or laotian* or latvia* or lebanon* or lebanese* or lesotho* or lesothan* or lesothonian* or basutoland* or mosotho* or basotho* or liberia* or libya* or jamahiriya* or lithuania* or macedonia* or madagasca* or malagasy* or malawi* or nyasaland* or malaysia* or malay* federation or maldives* or maldivian* or indian ocean or mali or malian* or "mali's" or malta or maltese* or "malta's" or micronesia* or marshallese* or kiribati* or marshall island* or nauru or nauran or nauruans or "naurian's" or mariana or marianas or palau or paluan* or tuvalu* or mauritania* or mauritan* or mauritius* or mexico* or mexican* or moldova* or moldovia* or mongol* or montenegr* or morocco* or moroccan* or ifni or mozambique* or mozambican* or myanmar* or burma* or burmese or namibia* or nepal* or new caledonia* or netherlands antill* or nicaragua* or niger* or oman or omani or omanis or "oman's" or pakistan* or palestin* or gaza* or west bank* or panama* or paraguay* or peru or peruvian* or "peru's" or philippine* or philipine* or phillipine* or phillippine* or filipino* or filipina* or poland* or polish or pole or poles or portugal* or portuguese or puerto ric* or romania* or russia* or ussr* or soviet* or rwanda* or rwandese or ruanda* or ruandese or samoa* or navigator island* or pacific island* or polynesia* or "sao tome and principe*" or sao tomean* or santomean* or saudi arabia* or saudi or saudis or senegal* or serbia* or seychell* or sierra leone* or slovak* or sloven* or melanesia* or solomon island* or norfolk island* or somali* or sri lanka* or ceylon* or "saint kitts and nevis*" or "st kitts and nevis*" or kittian* or nevisian* or saint lucia* or st lucia* or saint vincent* or st vincent* or vincentian* or grenadine* or sudan* or surinam* or syria* or tajik* or tadjik* or tadzhik* or tanzania* or tanganyika* or thai* or timor leste* or east timor* or timorese* or togo or togoles* or "togo's" or tonga* or trinidad* or tobago* or tunisia* or turkiy* or turkey* or turk or turks or turkish or turkmen* or uganda* or ukrain* or uruguay* or uzbek* or vanuatu* or new hebrides* or venezuela* or vietnam* or viet nam* or yemen* or yugoslav* or zambia* or zimbabwe* or rhodesia* or arab* countr* or middle east* or global south or sahara* or subsahara* or magreb* or maghrib* or west indies* or caribbean* or central america* or latin america* or south america* or central asia* or north asia* or northern asia* or southeastern asia* or south eastern asia* or southeast asia* or south east asia* or west asia* or western asia* or east europe* or eastern europe* or developing countr* or developing nation* or developing population* or developing world or less developed countr* or less developed nation* or less developed world or lesser developed countr* or lesser developed nation* or lesser developed world or under developed countr* or under developed nation* or under developed world or underdeveloped countr* or underdeveloped nation* or underdeveloped world or middle income countr* or middle income

	nation* or middle income population* or low income countr* or low income nation* or low income population* or lower income countr* or lower income nation* or lower income population* or underserved countr* or underserved nation* or underserved population* or under served population* or under served nation* or under served population* or deprived countr* or deprived population* or high burden countr* or high burden nation* or countdown countr* or countdown nation* or poor countr* or poor nation* or poor population* or poor world or poorer countr* or poorer nation* or poorer population* or poorer world or developing econom* or less developed econom* or underdeveloped econom* or under developed econom* or middle income econom* or low income econom* or lower income econom* or low gdp or low gnp or low gross domestic or low gross national or lower gdp or lower gnp or lower gross domestic or lower gross national or lmic or lmics or third world or lami countr* or transitional countr* or emerging econom* or emerging nation*).ti,ab,hw,kf.
144.	15 or 143
145.	48 and 142 and 144
146.	(exp animal/ or exp invertebrate/ or nonhuman/ or animal experiment/ or animal tissue/ or animal model/ or exp plant/ or exp fungus/) not (exp human/ or human tissue/)
147.	145 not ((exp animal/ or exp invertebrate/ or nonhuman/ or animal experiment/ or animal tissue/ or animal model/ or exp plant/ or exp fungus/) not (exp human/ or human tissue/))
148.	limit 147 to yr="2000 -Current"

Table S2. Medline search strategy

1.	asia, southern/
2.	Middle East/
3.	exp Iran/
4.	exp Bahrain/
5.	exp Iraq/
6.	exp Jordan/
7.	exp Kuwait/
8.	exp Lebanon/
9.	exp Oman/
10.	exp Qatar/
11.	exp Saudi Arabia/
12.	exp Syria/
13.	exp United Arab Emirates/
14.	exp Yemen/
15.	exp "Democratic People's Republic of Korea"/
16.	exp Myanmar/
17.	indonesia/
18.	thailand/
19.	timor-leste/

20. afghanistan/
21. bangladesh/
22. bhutan/
23. india/
24. maldives/
25. nepal/
26. pakistan/
27. sri lanka/
28. "Eastern Mediterranean Region*".mp.
29. "South-East Asia Region*".mp.
30. "Middle East".mp. [mp=title, book title, abstract, original title, name of substance word, subject heading word, floating sub-heading word, keyword heading word, organism supplementary concept word, protocol supplementary concept word, rare disease supplementary concept word, unique identifier, synonyms, population supplementary concept word, anatomy supplementary concept word]
31. "South* Asia".mp. [mp=title, book title, abstract, original title, name of substance word, subject heading word, floating sub-heading word, keyword heading word, organism supplementary concept word, protocol supplementary concept word, rare disease supplementary concept word, unique identifier, synonyms, population supplementary concept word, anatomy supplementary concept word]
32. "Southeast Asia".mp. [mp=title, book title, abstract, original title, name of substance word, subject heading word, floating sub-heading word, keyword heading word, organism supplementary concept word, protocol supplementary concept word, rare disease supplementary concept word, unique identifier, synonyms, population supplementary concept word, anatomy supplementary concept word]
33. "South east Asia".mp. [mp=title, book title, abstract, original title, name of substance word, subject heading word, floating sub-heading word, keyword heading word,

	organism supplementary concept word, protocol supplementary concept word, rare disease supplementary concept word, unique identifier, synonyms, population supplementary concept word, anatomy supplementary concept word]
34.	(Iran or Bahrain or Iraq or Jordan or Kuwait or Lebanon or Oman or Qatar or "Saudi Arabia" or Syria or "United Arab Emirates" or Yemen or "Democratic People's Republic of Korea" or Myanmar or Indonesia or Thailand or "Timor-leste" or Afghanistan or Bangladesh or Bhutan or India or Maldives or Nepal or Pakistan or "Sri Lanka" or Palestine or "West Bank" or "Gaza").mp. [mp=title, book title, abstract, original title, name of substance word, subject heading word, floating sub-heading word, keyword heading word, organism supplementary concept word, protocol supplementary concept word, rare disease supplementary concept word, unique identifier, synonyms, population supplementary concept word, anatomy supplementary concept word]
35.	(Iranian* or Bahraini* or Iraqi* or Jordanian* or Kuwaiti* or Lebanese or Omani* or Qatari* or "Emirati*" or Syrian* or "Saudi Arabian*" or saudi* or Yemeni* or "Democratic People's Republic of Korea*" or Burmese or Myanma or Indonesian* or Thai or Timorese or Afghani* or Bengali* or Bhutanese or Indian* or Maldivian* or Nepalese or Nepali* or Pakistani* or "Sri Lankan" or Palestinian* or "Gazan*").mp. [mp=title, book title, abstract, original title, name of substance word, subject heading word, floating sub-heading word, keyword heading word, organism supplementary concept word, protocol supplementary concept word, rare disease supplementary concept word, unique identifier, synonyms, population supplementary concept word, anatomy supplementary concept word]
36.	exp Climate Change/
37.	"Climate change".mp. [mp=title, book title, abstract, original title, name of substance word, subject heading word, floating sub-heading word, keyword heading word, organism supplementary concept word, protocol supplementary concept word, rare disease supplementary concept word, unique identifier, synonyms, population supplementary concept word, anatomy supplementary concept word]
38.	("heavy rainfall*" or ((chang* or shift*) adj2 weather) or "weather pattern?" or "rising sea level?").mp. [mp=title, book title, abstract, original title, name of substance word, subject heading word, floating sub-heading word, keyword heading word, organism supplementary concept word, protocol supplementary concept word, rare disease

	supplementary concept word, unique identifier, synonyms, population supplementary concept word, anatomy supplementary concept word]
39.	((Glacier* or glacial* or iceberg* or "sea ice") adj2 (melt* or shrink* or sublimat*)).mp. [mp=title, book title, abstract, original title, name of substance word, subject heading word, floating sub-heading word, keyword heading word, organism supplementary concept word, protocol supplementary concept word, rare disease supplementary concept word, unique identifier, synonyms, population supplementary concept word, anatomy supplementary concept word]
40.	"Hot Temperature"/ or "Extreme Heat"/
41.	("Heat -related" or "hot weather" or "extreme heat" or "Extreme temperature?" or "Extreme Hot Weather" or "heat wave" or heatwave or "high temperature?" or "rising temperature?" or "extreme cold").mp. [mp=title, book title, abstract, original title, name of substance word, subject heading word, floating sub-heading word, keyword heading word, organism supplementary concept word, protocol supplementary concept word, rare disease supplementary concept word, unique identifier, synonyms, population supplementary concept word, anatomy supplementary concept word]
42.	*natural disasters/ or *avalanches/ or *cyclonic storms/ or *droughts/ or *floods/ or *landslides/ or *tornadoes/ or *wildfires/
43.	exp Extreme Weather/
44.	("severe weather" or "extreme weather" or Drought? or Flood* or "natural disaster?" or Hurricane* or Wildfire* or wild-fire* or tornado* or "Tropical storm" or avalanche? or cyclone* or landslide? or "land slide" or mudslide? or "mud slide?" or storm? or tornado* or typhoon?).mp. [mp=title, book title, abstract, original title, name of substance word, subject heading word, floating sub-heading word, keyword heading word, organism supplementary concept word, protocol supplementary concept word, rare disease supplementary concept word, unique identifier, synonyms, population supplementary concept word, anatomy supplementary concept word]
45.	(((emergency or emergencies) adj5 (environ* or human or manmade or "man made" or nature or natural or weather or respons*)) or ("heavy rainfall*" or ((chang* or shift*) adj2 weather) or "weather pattern?" or "rising sea level?")).mp. [mp=title, book title, abstract, original title, name of substance word, subject heading word, floating sub-heading word, keyword heading word, organism supplementary concept word, protocol supplementary concept word, rare disease supplementary concept word,

	unique identifier, synonyms, population supplementary concept word, anatomy supplementary concept word]
46.	(("Climate change*" or "climate change-related" or "climate-related") adj2 (windspeed? or "wind speed?" or "global stilling" or precipitation* or humid*)).mp. [mp=title, book title, abstract, original title, name of substance word, subject heading word, floating sub-heading word, keyword heading word, organism supplementary concept word, protocol supplementary concept word, rare disease supplementary concept word, unique identifier, synonyms, population supplementary concept word, anatomy supplementary concept word]
47.	(("Climate change*" or "climate change-related" or "climate-related") and (distribut* or spread* or transmi* or vector?) and (pathogen* or disease? or "infectious disease?" or virus* or bacteria*)).mp. [mp=title, book title, abstract, original title, name of substance word, subject heading word, floating sub-heading word, keyword heading word, organism supplementary concept word, protocol supplementary concept word, rare disease supplementary concept word, unique identifier, synonyms, population supplementary concept word, anatomy supplementary concept word]
48.	(exp Climate Change/ or exp Extreme Weather/) and (*communicable diseases/ or *blood-borne infections/ or *communicable diseases, emerging/ or *communicable diseases, imported/ or exp Insect Vectors/)
49.	exp Greenhouse Effect/
50.	exp Greenhouse Gases/
51.	((("greenhouse effect" or GHG? or Greenhouse Gas* or "carbon dioxide" or "CO2" or "CO2 equivalent*" or "nitrous oxide" or CH4 or "Sulphur dioxide" or "Sulfur dioxide" or "SO2" or "NO2" or "combustion air pollution" or Chlorofluorocarbon* or trifluoride* or Methane or ammonia or nitrogen or CFC? or "nitrogen trifluoride*" or NF3 or Hydrofluorocarbon* or perfluorocarbon* or "sulfur hexafluoride" or "sulphur hexafluoride" or SF6) adj2 (increas* or level? or Atmospher* or emission*)) or "global warming" or (environmental adj degrad*)).mp. [mp=title, book title, abstract, original title, name of substance word, subject heading word, floating sub-heading word, keyword heading word, organism supplementary concept word, protocol supplementary concept word, rare disease supplementary concept word, unique identifier, synonyms, population supplementary concept word, anatomy supplementary concept word]

52.	(climate change/ and Air Pollution/) or (("Climate change*" or "climate change-related" or "climate-related") and (air adj2 (quality or pollut*))).mp.
53.	Water Quality/ or ((water or groundwater) adj (ground or quality or potab* or safe* or drink* or suppl* or secur* or insecur*)).mp. or ((Saltwater or "salt water" or "sea water") adj2 encroach*).mp. or (Salinat* adj2 (water or soil)).mp. [mp=title, book title, abstract, original title, name of substance word, subject heading word, floating sub-heading word, keyword heading word, organism supplementary concept word, protocol supplementary concept word, rare disease supplementary concept word, unique identifier, synonyms, population supplementary concept word, anatomy supplementary concept word]
54.	((climat* or "climate-related" or "climate change-related" or environment* or "environmentally-induced") adj2 ("human security" or change or warming or emergenc* or hazard* or cris?s or refugee* or shock* or variabilit* or displac* or "forced migration" or (availab* adj3 (food* or water or food system*)) or agricultur* or famine* or war* or armed conflict* or political conflict* or violen*)).mp.
55.	(exp Climate Change/ or exp Extreme Weather/) and (Refugees/ or food supply/ or famine/ or *"warfare and armed conflicts"/ or *agriculture/ or *crop production/ or *farms/ or *armed conflicts/ or Water Supply/)
56.	(Humans/ and ("Puberty, Precocious"/ or puberty/ or "puberty, delayed"/ or "Sexual Maturation"/)) or Menarche/ or (menarche* or "menstrual cycle" or menstruation).mp. [mp=title, book title, abstract, original title, name of substance word, subject heading word, floating sub-heading word, keyword heading word, organism supplementary concept word, protocol supplementary concept word, rare disease supplementary concept word, unique identifier, synonyms, population supplementary concept word, anatomy supplementary concept word]
57.	Sex Education/ or "family planning education".mp. or "family planning instructor*".mp. or "family planning training".mp. or "contraceptive counselling".mp. or "family planning programs".mp. or "sex education".mp. or "sex instruction".mp. or "sexual health".mp. or "menstrual hygiene".mp. [mp=title, book title, abstract, original title, name of substance word, subject heading word, floating sub-heading word, keyword heading word, organism supplementary concept word, protocol supplementary concept word, rare disease supplementary concept word, unique identifier, synonyms, population supplementary concept word, anatomy supplementary concept word]

58.	Infertility/ or "Reproductive Sterility".mp. or sterility.mp. or Subfertility.mp. or Sub-Fertility.mp. or "female infertility".mp. or "male infertility".mp. or infertil*.mp. [mp=title, book title, abstract, original title, name of substance word, subject heading word, floating sub-heading word, keyword heading word, organism supplementary concept word, protocol supplementary concept word, rare disease supplementary concept word, unique identifier, synonyms, population supplementary concept word, anatomy supplementary concept word]
59.	Contraceptives, Postcoital/ or contraception/ or coitus interruptus/ or contraception, barrier/ or contraception, postcoital/ or natural family planning methods/ or Infertility/
60.	exp Condoms/
61.	exp Contraceptive agents/ or exp Contraceptive devices/
62.	exp Sexual Dysfunction, Physiological/ or exp Sexual Dysfunctions, Psychological/
63.	("birth regulation" or "birth control*" or "coitus interruptus" or "fertility control" or contracept* or "unprotected sex" or "unsafe sex" or antifertility or anticonception or "fertility control" or "fertilization inhibition" or "inhibition of fertilization" or "fertilisation inhibition" or "inhibition of fertilisation" or "morning after pill" or "postcoital antifertility agent" or "postcoital pill").mp. [mp=title, book title, abstract, original title, name of substance word, subject heading word, floating sub-heading word, keyword heading word, organism supplementary concept word, protocol supplementary concept word, rare disease supplementary concept word, unique identifier, synonyms, population supplementary concept word, anatomy supplementary concept word]
64.	("cervical cap" or "cervical caps" or "cervix cap" or "dalkon shield" or "dana deveice" or "i.u.d." or "intra uterine device*" or "intracervical device" or "intrauterine device*" or "intrauterine devices" or "intravaginal pessaries" or "intravaginal pessary" or "iucd" or "iud" or "iuds" or "Lippes loop" or "marguilies spiral" or "margulie coil" or "progestin implant" or "vagina pessaries" or "vagina pessary" or "vaginal diaphragm" or "vaginal diaphragms" or "vaginal pessaries" or "vaginal pessary" or "vaginal ring" or "vaginal shield" or "vaginal rings" or "vaginal shields" or "vaginal sponge" or "vaginal sponges" or condom or condom*).mp. [mp=title, book title, abstract, original title, name of substance word, subject heading word, floating sub-heading word, keyword heading word, organism supplementary concept word, protocol supplementary concept word, rare disease supplementary concept word, unique identifier, synonyms, population supplementary concept word, anatomy supplementary concept word]

65.	abortion, induced/ or Abortion, Criminal/ or abortion, eugenic/ or abortion, legal/ or abortion, therapeutic/ or Abortion, Septic/ or (abortion* or abortus).mp. or Misoprostol/ or Mifepristone/
66.	Birth Intervals/ or ("birth interval" or "birth intervals" or "birth spacing" or "birth spacings" or "child spacing" or "child spacings" or "family building" or "family planning" or "pregnancy interval" or "pregnancy intervals").mp.
67.	Gynecology/ or (gynaecology or gynecology).mp. or Reproductive Health/ or "reproductive health".mp. [mp=title, book title, abstract, original title, name of substance word, subject heading word, floating sub-heading word, keyword heading word, organism supplementary concept word, protocol supplementary concept word, rare disease supplementary concept word, unique identifier, synonyms, population supplementary concept word, anatomy supplementary concept word]
68.	Maternal Health/ or Maternal Welfare/ or Obstetrics/ or ("maternal health maternal welfare" or "maternal child health" or "maternal child welfare").mp. or obstetric*.mp. or "minimum initial service package".mp. or "maternal welfare".mp. [mp=title, book title, abstract, original title, name of substance word, subject heading word, floating sub-heading word, keyword heading word, organism supplementary concept word, protocol supplementary concept word, rare disease supplementary concept word, unique identifier, synonyms, population supplementary concept word, anatomy supplementary concept word]
69.	Maternal Mortality/
70.	Midwifery/ or ("birth attendant" or "birth attendants" or midwife or midwives or midwifery).mp. or Postnatal Care/ or Postpartum Period/ or (postnatal or "post natal" or postpartum or "post partum" or puerperium or puerperal).mp.
71.	*Pregnancy/ or "child bearing".mp. or childbearing.mp. or Prenatal Care/ or ("prenatal" or "pre natal" or "antenatal" or "ante natal").mp. or Perinatal Care/ or ("perinatal care" or "peri natal care" or "perinatal care" or "peri natal care").mp. or Peripartum Period/ or ("peripartum* period*" or "perinatal* period*" or "peri natal* period*").mp. or Parturition/
72.	(birth or childbirth* or parturition* or "safe delivery" or "safely delivered" or (antepartum or "ante partum" or intrapartum or "intra partum")).mp.

73.	Stillbirth/ or Abortion, Spontaneous/ or (stillbirth* or stillborn* or "still birth*" or "still born*" or "still born*" or miscarriage*).mp. [mp=title, book title, abstract, original title, name of substance word, subject heading word, floating sub-heading word, keyword heading word, organism supplementary concept word, protocol supplementary concept word, rare disease supplementary concept word, unique identifier, synonyms, population supplementary concept word, anatomy supplementary concept word]
74.	("abnormal labor" or "delayed labor" or "delayed labour" or "inertia uteri" or "labor obstruction" or "labour obstruction" or "obstructed labor" or "obstructed labour" or "uterus inertia" or dystocia or dystocias).mp. or Breech Presentation/ or (breech adj2 (present* or position*)).mp. or Uterine Hemorrhage/ or ("vagina* haemorrhage" or "vagina* hemorrhage" or "vaginal bleeding").mp.
75.	("Social Discrimination"/ or "Social Stigma"/) and ("Sexual and Gender Minorities"/ or "Gender Identity"/ or Transsexualism/)
76.	((discriminat* or stigma* or "social exclusion*" or persecut* or violen*) adj3 (Non-Heterosexual? or GLBT or LGBTQ or "Gender Divers*" or LBG or LGBT or "Sexual Minorit*" or LGBTQIA or Bisexual* or lesbian* or gay* or homosexual* or Queer* or "Men Who Have Sex With Men" or MSM or "Women Who Have Sex With Women" or WSW or transgender* or transsexual* or trans-gender* or Transmen or transman or trans-sexual* or intersex* or inter-sex* or 2spirit* or twospirit* or "2 Spirit*" or "two spirit*" or trans-women or trans-woman or "Gender Minorit*")).mp.
77.	Gender Equity/
78.	Sex Workers/
79.	Women's Rights/
80.	(gender adj3 (equalit* or equity or identit* or norm? or role or violence)).mp. [mp=title, book title, abstract, original title, name of substance word, subject heading word, floating sub-heading word, keyword heading word, organism supplementary concept word, protocol supplementary concept word, rare disease supplementary concept word, unique identifier, synonyms, population supplementary concept word, anatomy supplementary concept word]
81.	(women* adj2 (liberation or right* or status)).mp. [mp=title, book title, abstract, original title, name of substance word, subject heading word, floating sub-heading word, keyword heading word, organism supplementary concept word, protocol

	supplementary concept word, rare disease supplementary concept word, unique identifier, synonyms, population supplementary concept word, anatomy supplementary concept word]
82.	*hiv infections/ or *acquired immunodeficiency syndrome/ or ("aids" or "acquired immune deficiency syndrome*" or "acquired immuno deficiency syndrome* acquired immunodeficiency syndrome*").mp. or Infectious Disease Transmission, Vertical/pc or (pmtct or "prevention of HIV mother to child transmission" or "prevention of mother to child transmission" or "eliminate mother to child transmission" or "prevention of mother to child HIV transmission").mp. or "human immunodeficiency virus".mp. or HIV.mp. [mp=title, book title, abstract, original title, name of substance word, subject heading word, floating sub-heading word, keyword heading word, organism supplementary concept word, protocol supplementary concept word, rare disease supplementary concept word, unique identifier, synonyms, population supplementary concept word, anatomy supplementary concept word]
83.	Sexually Transmitted Diseases/ or ("sexually transmitted disease*" or "sexually transmitted infection*" or "venereal disease*" or "venereal infection" or "venereal infections").mp. or "sexually transmitted".mp. or STI*.mp. or STD*.mp. or sexually transmitted diseases, bacterial/ or chancroid/ or chlamydia infections/ or lymphogranuloma venereum/ or gonorrhea/ or syphilis/ or herpes genitalis/ or Condylomata Acuminata/ [mp=title, book title, abstract, original title, name of substance word, subject heading word, floating sub-heading word, keyword heading word, organism supplementary concept word, protocol supplementary concept word, rare disease supplementary concept word, unique identifier, synonyms, population supplementary concept word, anatomy supplementary concept word]
84.	("calymmatobacterium granulomatis infection" or "chancroid" or "chancroids" or chlamidiosis or "chlamydia infection" or "chlamydia infections" or "Donovania granulomatis infection" or "donovanosis" or "frei disease" or "gonococcal infection" or "gonococcal infections" or "gonococcosis" or "gonococcus or infection" or "gonorrhea" or "gonorrheas" or "gonorrhoea" or "granuloma inguinale" or "granuloma or venereum" or "great pox" or "inguinal lymphogranulomatosis" or "Klebsiella granulomatis infection" or "lues" or "lymphogranuloma inguinale" or "lymphogranuloma venereum" or "lymphogranuloma venerum" or "lymphopathia venerea" or "lymphopathia venereum" or "Neisseria gonorrhoeae infection" or "nicholas favre disease" or "nicolas favre disease" or "syphilis" or "syphilitic disorder" or "venereal lymphogranuloma" or "HPV" or "human papilloma virus" or "human papillomavirus").mp. [mp=title, book title, abstract, original title, name of substance word, subject heading word, floating sub-heading word, keyword heading word,

	organism supplementary concept word, protocol supplementary concept word, rare disease supplementary concept word, unique identifier, synonyms, population supplementary concept word, anatomy supplementary concept word]
85.	("anal wart" or "anal warts" or "anogenital wart" or "anogenital warts" or "condyla acuminatum" or "condylatum acuminatum" or "condyloma accuminatum" or "condyloma acuminata" or "condylomata acuminata" or "genital herpes" or "genital wart" or "genital warts" or "herpes genitalis" or "herpes or progenitalis" or "herpes simplex genitalis" or "herpes simplex virus genital infection" or "penile wart" or "penile warts" or "perianal wart" or "perianal warts" or "venereal wart" or "venereal warts" or "verruca or accuminata" or "vulvar condyloma").mp. or Scabies/ or ("sarcoptic mange" or scabies).mp. or Phthirus/ or ("crab lice" or "crab lices" or "crab louse" or "crab louses" or "Pediculus pubis" or "phthirus" or "Phtirus pubis" or "Pthirus pubis" or "pubic lice" or "pubic louse").mp. [mp=title, book title, abstract, original title, name of substance word, subject heading word, floating sub-heading word, keyword heading word, organism supplementary concept word, protocol supplementary concept word, rare disease supplementary concept word, unique identifier, synonyms, population supplementary concept word, anatomy supplementary concept word]
86.	Intimate Partner Abuse/ or Spouse abuse/ or Domestic Violence/ or "Gender-Based Violence"/ or *"Honor-Based Violence"/ or Circumcision, Female/ or ("partner abuse" or "partner violence" or "wife abuse" or "spouse abuse" or "spousal abuse" or "domestic violence" or "domestic abuse" or "gender based violence" or GBV or "violence against women and girls" or "sex* based violence" or "Dowry Death?" or hono?r killing* or "Hono?r Based Violence" or "Hono?r Crime*").mp. or ((abuse* or assault* or violence) adj2 (woman or women)).mp. or Sex offenses/ or Human Trafficking/ or Rape/ or ("coerced intercourse" or "forced prostitution" or "forced sex" or "human trafficking" or "human traffickings" or rape* or "sex trafficking" or "sex traffickings" or "sex* abuse*" or "sex* assault*" or "sex* crime*" or "sex* offense" or "sex* offenses" or "sex* slave*" or "sexual aggression" or "sexual bullying" or "sexual coercion" or "sexual exploitation*" or "sexual harassment" or "sexual trauma" or "sexual violence" or "emotional violence" or IPV or FGM or "intimate partner violence" or "female genital mutilation" or female circumcision or Clitoridectom* or Clitorectom* or Infibulation? or "genital cutting" or "child marriage" or femicide or fillicide).mp. or ("physical* abuse*" or "physical* assault*" or "physical violence").mp. or fistula/ or vaginal fistula/ or rectovaginal fistula/ or (fistula or fistulas or "genital trauma" or "genital injury" or "vaginal trauma" or "vaginal injury").mp.
87.	exp Intimate Partner Violence/

88.	((child or early or forced) adj marriage).mp. [mp=title, book title, abstract, original title, name of substance word, subject heading word, floating sub-heading word, keyword heading word, organism supplementary concept word, protocol supplementary concept word, rare disease supplementary concept word, unique identifier, synonyms, population supplementary concept word, anatomy supplementary concept word]
89.	exp Infant, Newborn/
90.	36 or 37 or 38 or 39 or 40 or 41 or 42 or 43 or 44 or 45 or 46 or 47 or 48 or 49 or 50 or 51 or 52 or 53 or 54 or 55
91.	56 or 57 or 58 or 59 or 60 or 61 or 62 or 63 or 64 or 65 or 66 or 67 or 68 or 69 or 70 or 71 or 72 or 73 or 74 or 75 or 76 or 77 or 78 or 79 or 80 or 81 or 82 or 83 or 84 or 85 or 86 or 87 or 88 or 89
92.	(afghan* or africa* or albania* or algeria* or angola* or antigua* or barbuda* or argentin* or armenia* or aruba* or azerbaijan* or bahrain* or bangladesh* or bengal* or bangal* or barbados* or barbadian* or bajan or bajans or belarus* or belorus* or byelarus* or byelorus* or belize* or benin* or dahomey or bhutan* or bolivia* or bosnia* or herzegovin* or botswan* or batswan* or bechuanaland* or brazil* or brasil* or bulgaria* or burkina* or burkinese* or upper volta* or burundi* or urundi* or cabo verde* or cape verde* or cambodia* or kampuchea* or khmer* or cameroon* or cameroun* or ubangi shari* or chad* or chile* or china* or chinese or colombia* or comoro* or comore* or comorian* or mayotte* or congo* or zaire* or costa rica* or "cote d'ivoir*" or "cote d' ivoir*" or cote divoir* or cote d ivoir* or ivory coast* or ivorian* or croatia* or cuba or cuban or cubans or "cuba's" or cyprus* or cypriot* or czech* or djibouti* or french somaliland* or dominica* or ecuador* or egypt* or united arab republic* or el salvador* or salvadoran* or guinea* or equatoguinea* or eritrea* or estonia* or eswatini* or swaziland* or swazi* or swati* or ethiopia* or fiji* or gabon* or gabonese* or gabonaise* or gambia* or ((georgia or georgian or georgians) not (atlanta or california or florida)) or ghana* or gibraltar* or greece* or greek* or grecian* or grenada* or grenadian* or guam* or guatemala* or guyana* or guiana* or guyanese* or haiti* or hispaniola* or hondura* or hungary* or hungarian* or india* or indonesia* or iran* or iraq* or isle of man* or jamaica* or jordan* or kazakh* or kenya* or karabati* or korea* or kosovo* or kosova* or kyrgyz* or kirgiz* or kirghiz* or laos or lao or laotian* or latvia* or lebanon* or lebanese* or lesotho* or lesothan* or lesothonian* or basutoland* or mosotho* or basotho* or liberia* or libya* or jamahiriya* or lithuania* or macedonia* or madagasca* or malagasy* or malawi* or nyasaland* or malaysia* or malay* federation or maldives* or maldivian* or indian ocean or mali or malian* or "mali's" or malta or maltese* or "malta's" or micronesia* or marshallese* or

kiribati* or marshall island* or nauru or nauran or nauruans or "naurian's" or mariana or marianas or palau or paluan* or tuvalu* or mauritania* or mauritan* or mauritius* or mexico* or mexican* or moldova* or moldovia* or mongol* or montenegr* or morocco* or moroccan* or ifni or mozambique* or mozambican* or myanmar* or burma* or burmese or namibia* or nepal* or new caledonia* or netherlands antill* or nicaragua* or niger* or oman or omani or omanis or "oman's" or pakistan* or palestin* or gaza* or west bank* or panama* or paraguay* or peru or peruvian* or "peru's" or philippine* or philipine* or phillipine* or phillippine* or filipino* or filipina* or poland* or polish or pole or poles or portugal* or portuguese or puerto ric* or romania* or russia* or ussr* or soviet* or rwanda* or rwandese or ruanda* or ruandese or samoa* or navigator island* or pacific island* or polynesia* or "sao tome and principe*" or sao tomean* or santomean* or saudi arabia* or saudi or saudis or senegal* or serbia* or seychell* or sierra leone* or slovak* or sloven* or melanesia* or solomon island* or norfolk island* or somali* or sri lanka* or ceylon* or "saint kitts and nevis*" or "st kitts and nevis*" or kittian* or nevisian* or saint lucia* or st lucia* or saint vincent* or st vincent* or vincentian* or grenadine* or sudan* or surinam* or syria* or tajik* or tadjik* or tadzhik* or tanzania* or tanganyika* or thai* or timor leste* or east timor* or timorese* or togo or togoles* or "togo's" or tonga* or trinidad* or tobago* or tunisia* or turkiy* or turkey* or turk or turks or turkish or turkmen* or uganda* or ukrain* or uruguay* or uzbek* or vanuatu* or new hebrides* or venezuela* or vietnam* or viet nam* or yemen* or yugoslav* or zambia* or zimbabwe* or rhodesia* or arab* countr* or middle east* or global south or sahara* or subsahara* or magreb* or maghrib* or west indies* or caribbean* or central america* or latin america* or south america* or central asia* or north asia* or northern asia* or southeastern asia* or south eastern asia* or southeast asia* or south east asia* or west asia* or western asia* or east europe* or eastern europe* or developing countr* or developing nation* or developing population* or developing world or less developed countr* or less developed nation* or less developed world or lesser developed countr* or lesser developed nation* or lesser developed world or under developed countr* or under developed nation* or under developed world or underdeveloped countr* or underdeveloped nation* or underdeveloped world or middle income countr* or middle income nation* or middle income population* or low income countr* or low income nation* or low income population* or lower income countr* or lower income nation* or lower income population* or underserved countr* or underserved nation* or underserved population* or under served population* or under served nation* or under served population* or deprived countr* or deprived population* or high burden countr* or high burden nation* or countdown countr* or countdown nation* or poor countr* or poor nation* or poor population* or poor world or poorer countr* or poorer nation* or poorer population* or poorer world or developing econom* or less developed econom* or underdeveloped econom* or under developed econom* or middle income econom* or

	low income econom* or lower income econom* or low gdp or low gnp or low gross domestic or low gross national or lower gdp or lower gnp or lower gross domestic or lower gross national or lmic or lmics or third world or lami countr* or transitional countr* or emerging econom* or emerging nation*).ti,ab,hw,kf.
93.	1 or 2 or 3 or 4 or 5 or 6 or 7 or 8 or 9 or 10 or 11 or 12 or 13 or 14 or 15 or 16 or 17 or 18 or 19 or 20 or 21 or 22 or 23 or 24 or 25 or 26 or 27 or 28 or 29 or 30 or 31 or 32 or 33 or 34 or 35 or 92
94.	90 and 91 and 93
95.	exp animals/ not humans.sh.
96.	94 not 95
97.	limit 96 to yr="2000 -Current"

Text S1: Scopus search strategy

(((TITLE-ABS-KEY ("gender equity" OR "gender equality" OR "sex worker*" OR "women* rights" OR (gender W/3 (equalit* OR equity OR identit* OR norm? OR role OR violence)))) OR (TITLE-ABS-KEY ((discriminat* OR stigma* OR "social exclusion*" OR persecut* OR violen*) AND (non-heterosexual? OR glbt OR lgbtq OR "Gender Divers*" OR lbg OR lgbt OR "Sexual Minorit*" OR lgbtqia OR bisexual* OR lesbian* OR gay* OR homosexual* OR queer* OR "Men Who Have Sex With Men" OR msm OR "Women Who Have Sex With Women" OR wsw OR transgender* OR transsexual* OR trans-gender* OR transmen OR transman OR trans-sexual* OR intersex* OR inter-sex* OR 2spirit* OR two AND spirit* OR "2 Spirit*" OR "two spirit*" OR "trans-women" OR "trans-woman" OR "Gender Minorit*"))) OR ((TITLE-ABS-KEY ("low birth weight" OR "small for gestational age" OR ((child OR early OR forced) W/1 marriage) OR "child marriage" OR "girl bride" OR "girl child marriage")) OR (TITLE-ABS-KEY ("intimate partner violence" OR "partner abuse" OR "partner violence" OR "wife abuse" OR "spouse abuse" OR "spousal abuse" OR "domestic violence" OR "domestic abuse" OR "gender based violence" OR "gender-based violence" OR gbv OR "violence against women and girl*" OR "sex* violence" OR "Dowry Death?" OR "honor killing*" OR "honour killing" OR "Honour Based Violence" OR "Human Trafficking" OR rape* OR "coerced intercourse" OR "forced prostitution" OR "forced sex" OR "sex trafficking" OR "sex* abuse*" OR "sex* assault*" OR "sex* crime*" OR "sex* offense" OR "sex* offenses" OR "sex* slave*" OR "sexual aggression" OR "sexual bullying" OR "sexual coercion" OR "sexual exploitation*" OR "sexual harassment" OR "sexual trauma" OR "sexual violence" OR "emotional violence" OR "psychological violence" OR ipv OR fgm OR "female genital mutilation" OR "female circumcision" OR clitoridectom* OR clitorectom* OR infibulation? OR "genital cutting" OR "genital mutilation" OR femicide OR fillicide OR "physical* abuse*" OR "physical* assault*" OR "physical violence")) OR (TITLE-ABS-KEY ("anal wart" OR "anal warts" OR "anogenital wart" OR "anogenital warts" OR "condyla acuminatum" OR "condylatum acuminatum" OR "condylomaaccuminatum" OR "condyloma acuminata" OR "condylomata acuminata" OR "genital herpes" OR "genital wart" OR "genital warts" OR "herpes genitalis" OR "herpes or progenitalis" OR "herpes simplex genitalis" OR "herpes simplex virus genital infection" OR "penile wart" OR "penile warts" OR "perianalwart" OR "perianal warts" OR "venereal wart" OR "venereal warts" OR "verruca or accuminata" OR "vulvar condyloma" OR scabies OR "sarcopticmange" OR scabie OR "crab lice" OR "crab lices" OR "crab louse" OR "crab louses" OR "Pediculus pubis" OR "phthirus" OR "Phtiruspubis" OR "Pthirus pubis" OR "pubic lice" OR "pubic louse")) OR (TITLE-ABS-KEY ("calymmatobacterium granulomatis infection" OR "chancroid" OR "chancroids" OR chlamidiosis OR "chlamydia infection" OR "chlamydia infections" OR "Donovania granulomatis infection" OR "donovanosis" OR "frei disease" OR "gonococcal infection" OR "gonococcal infections" OR "gonococcosis" OR "gonococcus or infection" OR

"gonorrhea" OR "gonorrheas" OR "gonorrhoea" OR "granuloma inguinale" OR "granuloma or venereum" OR "great pox" OR "inguinal lymphogranulomatosis" OR "Klebsiella granulomatis infection" OR "lues" OR "lymphogranuloma inguinale" OR "lymphogranuloma venereum" OR "lymphogranuloma venerum" OR "lymphopathia venerea" OR "lymphopathia venereum" OR "Neisseria gonorrhoeae infection" OR "nicholas favredisease" OR "nicolas favre disease" OR "syphilis" OR "syphilitic disorder" OR "venereal lymphogranuloma" OR "HPV" OR "human papilloma virus" OR "human papillomavirus")) OR (TITLE-ABS-KEY ("sexually transmitted disease*" OR "sexually transmitted infection*" OR "venereal disease*" OR "venereal infection" OR "venereal infections" OR "sexually transmitted")) OR ("aids" OR "acquired immune deficiency syndrome*" OR "acquired immuno deficiency syndrome* acquired immunodeficiency syndrome*" OR pmtct OR "prevention of HIV mother to child transmission" OR "prevention of mother to child transmission" OR "eliminate mother to child transmission" OR "prevention of mother to child HIV transmission" OR "human immunodeficiency virus" OR hiv) OR (TITLE-ABS-KEY ((women* W/2 (liberation OR right* OR status)))) OR (TITLE-ABS-KEY (breech W/2 (present* OR position*))) OR ("Breech Presentation" OR "Uterine Hemorrhage" OR "vagina* haemorrhage" OR "vagina* hemorrhage" OR "vaginal bleeding") OR (TITLE-ABS-KEY ("abnormal labor" OR "delayed labor" OR "delayed labour" OR "inertia uteri" OR "labor obstruction" OR "labour obstruction" OR "obstructed labor" OR "obstructed labour" OR "uterus inertia" OR dystocia OR dystocias)) OR (TITLE-ABS-KEY (stillborn* OR miscarriage* OR "spontaneous abortion")) OR (TITLE-ABS-KEY (birth OR childbirth* OR parturition* OR "safe delivery" OR "safely delivered" OR antepartum OR "ante partum" OR intrapartum OR "intra partum")) OR (TITLE-ABS-KEY (("maternal mortality" OR "birth attendant" OR "birth attendants" OR midwife OR midwives OR midwifery OR "Pregnancy" OR "child bearing" OR childbearing OR "prenatal" OR "pre natal" OR "antenatal" OR "ante natal") OR ("perinatal care" OR "peri natal care" OR "perinatal care" OR "peri natal care" OR "peripartum* period*" OR "perinatal* period*" OR "peri natal* period*"))) OR (TITLE-ABS-KEY ("maternal health" OR "maternal welfare" OR "maternal child health" OR "maternal child welfare" OR obstetrics)) OR (TITLE-ABS-KEY (gynaecology OR gynecology)) OR (TITLE-ABS-KEY (abortion* OR "pregnancy termination" OR misoprostol OR mifepristone OR "birth interval*" OR "birth spacing" OR "child spacing" OR "pregnancy interval" OR "pregnancy spacing")) OR ("cervical cap" OR "cervical caps" OR "cervix cap" OR "dalkon shield" OR "dana deveice" OR "i.u.d." OR "intra uterine device*" OR "intracervical device" OR "intrauterine device*" OR "intrauterine devices" OR "intravaginal pessaries" OR "intravaginal pessary" OR "iucd" OR "iud" OR "iuds" OR "Lippes loop" OR "marguilies spiral" OR "margulie coil" OR "progestin implant" OR "vagina pessaries" OR "vagina pessary" OR "vaginal diaphragm" OR "vaginal diaphragms" OR "vaginal pessaries" OR "vaginal pessary" OR "vaginal ring" OR "vaginal shield" OR "vaginal rings" OR "vaginal shields" OR "vaginal

sponge" OR "vaginal sponges" OR condom OR condom*) OR (TITLE-ABS-KEY ("birth regulation" OR "birth control*" OR "coitus interruptus" OR "fertility control" OR "unprotected sex" OR "unsafe sex" OR antifertility OR anticonception OR "fertility control" OR "fertilization inhibition" OR "inhibition of fertilization" OR "fertilisation inhibition" OR "inhibition of fertilisation" OR "morning after pill" OR "postcoital antifertility agent" OR "postcoital pill")) OR (TITLE-ABS-KEY ("sexual dysfunction" OR "Desire Disorder" OR "Arousal Disorder" OR "Erectile Dysfunction" OR "Orgasmic Disorder" OR "reproductive medicine")) OR (TITLE-ABS-KEY (contraceptive* OR contraception OR condom*)) OR (TITLE-ABS-KEY (infertility OR "Reproductive Sterility" OR sterility)) OR (TITLE-ABS-KEY ("Sex Education" OR "family planning education" OR "family planning instructor*" OR "family planning training" OR "contraceptive counselling" OR "family planning programs" OR "sex education" OR "sex instruction" OR "sexual health" OR "menstrual hygiene")) OR (TITLE-ABS-KEY (puberty OR menarche OR "menstrual cycle" OR menstruation OR "sexual maturation")))) AND ((TITLE-ABS-KEY (afghanistan OR albania OR algeria OR "American Samoa" OR angola OR armenia OR azerbaijan OR bangladesh OR belarus OR byelarus OR belorussia OR belize OR benin OR bhutan OR bolivia OR bosnia OR botswana OR brazil OR bulgaria OR burma OR "Burkina Faso" OR burundi OR "Cabo Verde" OR "Cape Verde" OR cambodia OR cameroon OR "Central African Republic" OR chad OR china OR colombia OR comoros OR comores OR comoro OR congo OR "Costa Rica" OR "Cote DIvoire" OR cuba OR "Democratic People* Republic of Korea" OR djibouti OR dominica OR "Dominican Republic" OR ecuador OR egypt OR "El Salvador" OR eritrea OR ethiopia OR "Equatorial Guinea" OR fiji OR gabon OR gambia OR gaza OR "Georgia Republic" OR georgia OR ghana OR grenada OR grenadines OR guatemala OR guinea OR "Guinea Bissau" OR guyana OR haiti OR herzegovina OR hercegovina OR honduras OR india OR indonesia OR iran OR iraq OR "Ivory Coast" OR jamaica OR jordan OR kazakhstan OR kenya OR kiribati OR korea OR kosovo OR kyrgyz OR kirghizia OR kirghiz OR kyrgyzstan OR "Lao PDR" OR laos OR lebanon OR lesotho OR liberia OR libya OR macedonia OR madagascar OR malawi OR malay OR malaya OR malaysia OR maldives OR mali OR "Marshall Islands" OR mauritania OR mauritius OR mexico OR micronesia OR moldova OR mongolia OR montenegro OR morocco OR mozambique OR myanmar OR namibia OR nepal OR nicaragua OR niger OR nigeria OR pakistan OR palau OR papua AND new AND guinea OR paraguay OR peru OR philippines OR principe OR romania OR rwanda OR ruanda OR samoa OR "Sao Tome" OR senegal OR serbia OR "Sierra Leone" OR "Solomon Islands" OR somalia OR "South Africa" OR "South Sudan" OR "Sri Lanka" OR "St Lucia" OR "St Vincent" OR sudan OR surinam OR suriname OR swaziland OR syria OR "Syrian Arab Republic" OR tajikistan OR tadzhikistan OR tajikistan OR tadzhik OR tanzania OR thailand OR timor OR togo OR tonga OR tunisia OR turkey OR turkmen OR turkmenistan OR tuvalu OR uganda OR ukraine OR uzbek OR uzbekistan OR vanuatu OR venezuela OR

vietnam OR "West Bank" OR yemen OR zambia OR zimbabwe) OR TITLE-ABS-KEY ("Deprived Countries" OR "Deprived Population" OR "Deprived Populations" OR "Developing Countries" OR "Developing Country" OR "Developing Economies" OR "Developing Economy" OR "Developing Nation" OR "Developing Nations" OR "Developing Population" OR "Developing Populations" OR "Developing World" OR "LAMI Countries" OR "LAMI Country" OR "Less Developed Countries" OR "Less Developed Country" OR "Less Developed Economies" OR "Less Developed Nation" OR "Less Developed Nations" OR "Less Developed World" OR "Lesser Developed Countries" OR "Lesser Developed Nations" OR lmic OR lmics OR "Low GDP" OR "Low GNP" OR "Low Gross Domestic" OR "Low Gross National" OR "Low Income" OR "Lower income" OR "Lower GDP" OR "Lower Gross Domestic" OR "Middle Income" OR "Poor Countries" OR "Poor Country" OR "Poor Economies" OR "Poor Economy" OR "Poor Nation" OR "Poor Nations" OR "Poor Population" OR "Poor Populations" OR "poor world" OR "Poorer Countries" OR "Poorer Economies" OR "Poorer Economy" OR "Poorer Nations" OR "Poorer Population" OR "Poorer Populations" OR "Third World" OR "Transitional Countries" OR "Transitional Country" OR "Transitional Economies" OR "Transitional Economy" OR "Under Developed" OR "Under Served" OR "Underdeveloped Countries" OR "Underdeveloped Country" OR "underdeveloped economies" OR "underdeveloped nations" OR "underdeveloped population" OR "Underdeveloped World" OR "Underserved Countries" OR "Underserved Nations" OR "Underserved Population" OR "Underserved Populations")) AND ((("climate change" OR "heavy rain*" OR "sea level" OR "extreme heat" OR "rising temperature*" OR "extreme cold" OR "hot temperature" OR "heatwave" OR "heat wave" OR "natural disaster*" OR "severe weather" OR "extreme weather" OR drought? OR flood* OR hurricane* OR wildfire* OR wild-fire* OR tornado* OR "Tropical storm" OR avalanche? OR cyclone* OR landslide? OR "land slide" OR mudslide? OR "mud slide?" OR storm? OR tornado* OR typhoon? OR wind* OR precipitation OR humid* OR "greenhouse effect" OR "greenhouse gases" OR "water quality") OR ((water OR groundwater) W/1 (ground OR quality OR potab* OR safe* OR drink* OR suppl* OR secur* OR insecur*)) OR ((saltwater OR "salt water" OR "sea water") W/2 encroach*) OR (salinat* W/2 (water OR soil))) OR (TITLE-ABS-KEY (((glacier* OR glacial* OR iceberg* OR "sea ice") W/2 (melt* OR shrink* OR sublimat*)))) OR (TITLE-ABS-KEY (("climate change" AND ("infectious disease*" OR "communicable disease*" OR virus OR bacteria OR pathogen* OR epidemic* OR pandemic* OR outbreak*)))) OR (TITLE-ABS-KEY ("climate change" AND "air pollution")) OR (TITLE-ABS-KEY ("climate change" AND (refugee* OR "forced displacement" OR "forcibly displaced" OR food OR water OR agriculture OR famine OR war OR "armed conflict" OR "political conflict" OR violen*))))) AND PUBYEAR > 1999 AND PUBYEAR < 2026) AND NOT (((INDEXTERMS (animals OR animal)) AND NOT (INDEXTERMS (humans OR human)))))

Table S3. Web of science search strategy

#	Search Query	Database	Date Run
1	TS=((((((("climate change" AND (refugee* OR "forced displacement" OR "forcibly displaced" OR food OR water OR agriculture OR famine OR war OR "armed conflict" OR "political conflict" OR violen*)))) OR ((("climate change" AND "air pollution"))) OR ((("climate change" AND ("infectious disease*" OR "communicable disease*" OR virus OR bacteria OR pathogen* OR epidemic* OR pandemic* OR outbreak*))))) OR (title-abs KEY (((glacier* OR glacial* OR iceberg* OR "sea ice") NEAR/2 (melt* OR shrink* OR sublimat*))))) OR (("climate change" OR "heavy rain*" OR "sea level" OR "extreme heat" OR "rising temperature*" OR "extreme cold" OR "hot temperature" OR "heatwave" OR "heat wave" OR "natural disaster*" OR "severe weather" OR "extreme weather" OR drought? OR flood* OR hurricane* OR wildfire* OR wild-fire* OR tornado* OR "Tropical storm" OR avalanche? OR cyclone* OR landslide? OR "land slide" OR mudslide? OR "mud slide?" OR storm? OR tornado* OR typhoon? OR wind* OR precipitation OR humid* OR "greenhouse effect" OR "greenhouse gases" OR "water quality")) OR ((water OR groundwater) NEAR/1 (ground OR quality OR potab* OR safe* OR drink* OR suppl* OR secur* OR insecur*)) OR ((saltwater OR "salt water" OR "sea water") NEAR/2 encroach*) OR (salinat* NEAR/2 (water OR soil))))))	Web of Science Core Collection	Tue Jan 21 2025 14:44:42 GMT-0500 (Eastern Standard Time)

2	TS=(((((("low birth weight" OR "small for gestational age")) OR ((((child OR early OR forced) NEAR/1 marriage) OR "child marriage" OR "girl bride" OR "girl child marriage")) OR (("intimate partner violence" OR "partner abuse" OR "partner violence" OR "wife abuse" OR "spouse abuse" OR "spousal abuse" OR "domestic violence" OR "domestic abuse" OR "gender based violence" OR "gender-based violence" OR gbv OR "violence against women and girl*" OR "sex* violence" OR "Dowry Death?" OR "honor killing*" OR "honour killing" OR "Honour Based Violence" OR "Human Trafficking" OR rape* OR "coerced intercourse" OR "forced prostitution" OR "forced sex" OR "sex trafficking" OR "sex* abuse*" OR "sex* assault*" OR "sex* crime*" OR "sex* offense" OR "sex* offenses" OR "sex* slave*" OR "sexual aggression" OR "sexual bullying" OR "sexual coercion" OR "sexual exploitation*" OR "sexual harassment" OR "sexual trauma" OR "sexual violence" OR "emotional violence" OR "psychological violence" OR ipv OR fgm OR "female genital mutilation" OR "female circumcision" OR clitoridectom* OR clitorectom* OR infibulation? OR "genital cutting" OR "genital mutilation" OR femicide OR fillicide OR "physical* abuse*" OR "physical* assault*" OR "physical violence")) OR (("anal wart" OR "anal warts" OR "anogenital wart" OR "anogenital warts" OR "condyla acuminatum" OR "condylatum acuminatum" OR "condylomaaccuminatum" OR "condyloma acuminata" OR "condylomata acuminata" OR "genital herpes" OR "genital wart" OR "genital warts" OR "herpes genitalis" OR "herpes or progenitalis" OR "herpes simplex genitalis" OR "herpes simplex virus genital infection" OR "penile wart" OR "penile warts" OR "perianalwart" OR "perianal warts" OR "venereal wart" OR	Web of Science Core Collection	Tue Jan 21 2025 14:45:26 GMT-0500 (Eastern Standard Time)

"venereal warts" OR "verruca or accuminata" OR "vulvar condyloma" OR scabies OR "sarcopticmange" OR scabie OR "crab lice" OR "crab lices" OR "crab louse" OR "crab louses" OR "Pediculus pubis" OR "phthirus" OR "Phtiruspubis" OR "Pthirus pubis" OR "pubic lice" OR "pubic louse")) OR (("calymmatobacterium granulomatis infection" OR "chancroid" OR "chancroids" OR chlamidiosis OR "chlamydia infection" OR "chlamydia infections" OR "Donovania granulomatis infection" OR "donovanosis" OR "frei disease" OR "gonococcal infection" OR "gonococcal infections" OR "gonococcosis" OR "gonococcus or infection" OR "gonorrhea" OR "gonorrheas" OR "gonorrhoea" OR "granuloma inguinale" OR "granuloma or venereum" OR "great pox" OR "inguinal lymphogranulomatosis" OR "Klebsiella granulomatis infection" OR "lues" OR "lymphogranuloma inguinale" OR "lymphogranuloma venereum" OR "lymphogranuloma venerum" OR "lymphopathia venerea" OR "lymphopathia venereum" OR "Neisseria gonorrhoeae infection" OR "nicholas favredisease" OR "nicolas favre disease" OR "syphilis" OR "syphilitic disorder" OR "venereal lymphogranuloma" OR "HPV" OR "human papilloma virus" OR "human papillomavirus")) OR (("sexually transmitted disease*" OR "sexually transmitted infection*" OR "venereal disease*" OR "venereal infection" OR "venereal infections" OR "sexually transmitted")) OR ((("aids" OR "acquired immune deficiency syndrome*" OR "acquired immuno deficiency syndrome* acquired immunodeficiency syndrome*") OR (pmtct OR "prevention of HIV mother to child transmission" OR "prevention of mother to child transmission" OR "eliminate mother to

child transmission" OR "prevention of mother to child HIV transmission") OR "human immunodeficiency virus" OR hiv)) OR (((women* NEAR/2 (liberation OR right* OR status)))) OR (("gender equity" OR "gender equality" OR "sex worker*" OR "women's rights" OR (gender NEAR/3 (equalit* OR equity OR identit* OR norm? OR role OR violence)))) OR ((discriminat* OR stigma* OR "social exclusion*" OR persecut* OR violen*) AND (non-heterosexual? OR glbt OR lgbtq OR "Gender Divers*" OR lbg OR lgbt OR "Sexual Minorit*" OR lgbtqia OR bisexual* OR lesbian* OR gay* OR homosexual* OR queer* OR "Men Who Have Sex With Men" OR msm OR "Women Who Have Sex With Women" OR wsw OR transgender* OR transsexual* OR trans-gender* OR transmen OR transman OR trans-sexual* OR intersex* OR inter-sex* OR 2spirit* OR two AND spirit* OR "2 Spirit*" OR "two spirit*" OR "trans-women" OR "trans-woman" OR "Gender Minorit*")) OR (((breech NEAR/2 (present* OR position*)))) OR (("Breech Presentation" OR "Uterine Hemorrhage" OR "vagina* haemorrhage" OR "vagina* hemorrhage" OR "vaginal bleeding")) OR (("abnormal labor" OR "delayed labor" OR "delayed labour" OR "inertia uteri" OR "labor obstruction" OR "labour obstruction" OR "obstructed labor" OR "obstructed labour" OR "uterus inertia" OR dystocia OR dystocias)) OR ((stillborn* OR miscarriage* OR "spontaneous abortion")) OR ((birth OR childbirth* OR parturition* OR "safe delivery" OR "safely delivered" OR antepartum OR "ante partum" OR intrapartum OR "intra partum")) OR (("maternal mortality" OR "birth attendant" OR "birth attendants" OR midwife OR midwives OR midwifery OR "Pregnancy" OR "child bearing" OR childbearing OR "prenatal" OR "pre natal"

OR "antenatal" OR "ante natal") OR ("perinatal care" OR "peri natal care" OR "perinatal care" OR "peri natal care" OR "peripartum* period*" OR "perinatal* period*" OR "peri natal* period*")) OR (("maternal health" OR "maternal welfare" OR "maternal child health" OR "maternal child welfare" OR obstetrics)) OR ((gynaecology OR gynecology)) OR ((abortion* OR "pregnancy termination" OR misoprostol OR mifepristone OR "birth interval*" OR "birth spacing" OR "child spacing" OR "pregnancy interval" OR "pregnancy spacing")) OR (("cervical cap" OR "cervical caps" OR "cervix cap" OR "dalkon shield" OR "dana deveice" OR "i.u.d." OR "intra uterine device*" OR "intracervical device" OR "intrauterine device*" OR "intrauterine devices" OR "intravaginal pessaries" OR "intravaginal pessary" OR "iucd" OR "iud" OR "iuds" OR "Lippes loop" OR "marguilies spiral" OR "margulie coil" OR "progestin implant" OR "vagina pessaries" OR "vagina pessary" OR "vaginal diaphragm" OR "vaginal diaphragms" OR "vaginal pessaries" OR "vaginal pessary" OR "vaginal ring" OR "vaginal shield" OR "vaginal rings" OR "vaginal shields" OR "vaginal sponge" OR "vaginal sponges" OR condom OR condom*)) OR ((("birth regulation" OR "birth control*" OR "coitus interruptus" OR "fertility control" OR "unprotected sex" OR "unsafe sex" OR antifertility OR anticonception OR "fertility control" OR "fertilization inhibition" OR "inhibition of fertilization" OR "fertilisation inhibition" OR "inhibition of fertilisation" OR "morning after pill" OR "postcoital antifertility agent" OR "postcoital pill"))) OR (("sexual dysfunction" OR "Desire Disorder" OR "Arousal Disorder" OR "Erectile Dysfunction" OR "Orgasmic Disorder" OR "reproductive medicine")) OR ((contraceptive* OR contraception OR

condom*)) OR ((infertility OR "Reproductive Sterility" OR sterility)) OR (("Sex Education" OR "family planning education" OR "family planning instructor*" OR "family planning training" OR "contraceptive counselling" OR "family planning programs" OR "sex education" OR "sex instruction" OR "sexual health" OR "menstrual hygiene")) OR ((puberty OR menarche OR "menstrual cycle" OR menstruation OR "sexual maturation"))))))

3	TS=(Deprived Countries OR Deprived Population OR Deprived Populations OR Developing Countries OR Developing Country OR Developing Economies OR Developing Economy OR Developing Nation OR Developing Nations OR Developing Population OR Developing Populations OR Developing World OR LAMI Countries OR LAMI Country OR Less Developed Countries OR Less Developed Country OR Less Developed Economies OR Less Developed Nation OR Less Developed Nations OR Less Developed World OR Lesser Developed Countries OR Lesser Developed Nations OR LMIC OR LMICS OR Low GDP OR Low GNP OR Low Gross Domestic OR Low Gross National OR Low Income OR Lower GDP OR lower gross domestic OR Lower Income OR Middle Income OR Poor Countries OR Poor Country OR Poor Economies OR Poor Economy OR Poor Nation OR Poor Nations OR Poor Population OR Poor Populations OR poor world OR Poorer Countries OR Poorer Economies OR Poorer Economy OR Poorer Nations OR Poorer Population OR Poorer Populations OR Third World OR Transitional Countries OR Transitional Country OR Transitional Economies OR Transitional Economy OR Under Developed Countries OR Under Developed Country OR under developed nations OR Under Developed World OR Under Served Population OR Under Served Populations OR Underdeveloped Countries OR Underdeveloped Country OR underdeveloped economies OR underdeveloped nations OR underdeveloped population OR Underdeveloped World OR Underserved Countries OR Underserved Nations OR Underserved Population OR Underserved Populations)	Web of Science Core Collection	Tue Jan 21 2025 14:45:51 GMT-0500 (Eastern Standard Time)

4	TS=(Afghanistan OR Albania OR Algeria OR American Samoa OR Angola OR Armenia OR Azerbaijan OR Bangladesh OR Belarus OR Byelarus OR Belorussia OR Belize OR Benin OR Bhutan OR Bolivia OR Bosnia OR Botswana OR Brazil OR Bulgaria OR Burma OR Burkina Faso OR Burundi OR Cabo Verde OR Cape Verde OR Cambodia OR Cameroon OR Central African Republic OR Chad OR China OR Colombia OR Comoros OR Comores OR Comoro OR Congo OR Costa Rica OR Côte d'Ivoire OR Cuba OR Democratic People’s Republic of Korea OR Djibouti OR Dominica OR Dominican Republic OR Ecuador OR Egypt OR El Salvador OR Equatorial Guinea OR Eritrea OR Ethiopia OR Fiji OR Gabon OR Gambia OR Gaza OR Georgia OR Georgia Republic OR Ghana OR Grenada OR Grenadines OR Guatemala OR Guinea OR Guinea Bissau OR Guyana OR Haiti OR Herzegovina OR Hercegovina OR Honduras OR India OR Indonesia OR Iran OR Iraq OR Ivory Coast OR Jamaica OR Jordan OR Kazakhstan OR Kenya OR Kiribati OR Korea OR Kosovo OR Kyrgyz OR Kirghizia OR Kirghiz OR Kyrgyzstan OR Lao PDR OR Laos OR Lebanon OR Lesotho OR Liberia OR Libya OR Macedonia OR Madagascar OR Malawi OR Malay OR Malaya OR Malaysia OR Maldives OR Mali OR Marshall Islands OR Mauritania OR Mauritius OR Mexico OR Micronesia OR Moldova OR Mongolia OR Montenegro OR Morocco OR Mozambique OR Myanmar OR Namibia OR Nepal OR Nicaragua OR Niger OR Nigeria OR Pakistan OR Palau OR Papua New Guinea OR Paraguay OR Peru OR Philippines OR Principe OR Romania OR Rwanda OR Ruanda OR Samoa OR Sao Tome OR Senegal OR Serbia OR Sierra Leone OR Solomon Islands OR Somalia OR South Africa OR South Sudan OR Sri	Web of Science Core Collection	Tue Jan 21 2025 14:46:58 GMT-0500 (Eastern Standard Time)

	Lanka OR St Lucia OR St Vincent OR Sudan OR Surinam OR Suriname OR Swaziland OR Syria OR Syrian Arab Republic OR Tajikistan OR Tadzhikistan OR Tajikistan OR Tadzhik OR Tanzania OR Thailand OR Timor OR Togo OR Tonga OR Tunisia OR Turkey OR Turkmen OR Turkmenistan OR Tuvalu OR Uganda OR Ukraine OR Uzbek OR Uzbekistan OR Vanuatu OR Venezuela OR Vietnam OR West Bank OR Yemen OR Zambia OR Zimbabwe)		
5	#4 OR #3	Web of Science Core Collection	Tue Jan 21 2025 14:47:49 GMT-0500 (Eastern Standard Time)
6	#1 AND #2 AND #5	Web of Science Core Collection	Tue Jan 21 2025 14:47:55 GMT-0500 (Eastern Standard Time)
7	#1 AND #2 AND #5 Timespan: 2000-01-01 to 2025-01-21	Web of Science Core Collection	Tue Jan 21 2025 14:52:49 GMT-0500 (Eastern Standard Time)

8	TS=((animal* OR rat OR rats OR mouse OR mice OR murine OR dog OR dogs OR canine OR cat OR cats OR feline OR rabbit OR cow OR cows OR bovine OR rodent* OR sheep OR ovine OR pig OR swine OR porcine OR veterinar* OR chick* OR zebrafish* OR baboon* OR nonhuman* OR primate* OR cattle* OR goose OR geese OR duck OR macaque* OR avian* OR bird* OR fish*) NOT (human* OR patient* OR women OR woman OR men OR man))	Web of Science Core Collection	Tue Jan 21 2025 14:53:40 GMT-0500 (Eastern Standard Time)
9	#7 NOT #8 Timespan: 2020-01-01 to 2025-01-21	Web of Science Core Collection	Tue Jan 21 2025 14:54:41 GMT-0500 (Eastern Standard Time)
10	#7 NOT #8	Web of Science Core Collection	Tue Jan 21 2025 14:59:32 GMT-0500 (Eastern Standard Time)
11	#7 NOT #8	Web of Science Core Collection	Tue Jan 21 2025 15:01:06 GMT-0500 (Eastern Standard Time)

Text S2: Data extraction/Information charting

- The underlying data extraction and information charting have been publicly deposited and are available here: 10.6084/m9.figshare.33489616 (CC BY 4.0).
